# Whole-Genome Immunoinformatic Analysis of *F. tularensis*: Predicted CTL Epitopes Clustered in Hotspots Are Prone to Elicit a T-Cell Response

**DOI:** 10.1371/journal.pone.0020050

**Published:** 2011-05-20

**Authors:** Anat Zvi, Shahar Rotem, Erez Bar-Haim, Ofer Cohen, Avigdor Shafferman

**Affiliations:** Department of Biochemistry and Molecular Genetics, Israel Institute for Biological Research, Ness Ziona, Israel; Aston University, United Kingdom

## Abstract

The cellular arm of the immune response plays a central role in the defense against intracellular pathogens, such as *F. tularensis*. To date, whole genome immunoinformatic analyses were limited either to relatively small genomes (e.g. viral) or to preselected subsets of proteins in complex pathogens. Here we present, for the first time, an unbiased bacterial global immunoinformatic screen of the 1740 proteins of *F. tularensis* subs. holarctica (LVS), aiming at identification of immunogenic peptides eliciting a CTL response. The very large number of predicted MHC class I binders (about 100,000, IC_50_ of 1000 nM or less) required the design of a strategy for further down selection of CTL candidates. The approach developed focused on mapping clusters rich in overlapping predicted epitopes, and ranking these “hotspot” regions according to the density of putative binding epitopes. Limited by the experimental load, we selected to screen a library of 1240 putative MHC binders derived from 104 top-ranking highly dense clusters. Peptides were tested for their ability to stimulate IFNγ secretion from splenocytes isolated from LVS vaccinated C57BL/6 mice. The majority of the clusters contained one or more CTL responder peptides and altogether 127 novel epitopes were identified, of which 82 are non-redundant. Accordingly, the level of success in identification of positive CTL responders was 17–25 fold higher than that found for a randomly selected library of 500 predicted MHC binders (IC_50_ of 500 nM or less). Most proteins (ca. 2/3) harboring the highly dense hotspots are membrane-associated. The approach for enrichment of true positive CTL epitopes described in this study, which allowed for over 50% increase in the dataset of known T-cell epitopes of *F. tularensis*, could be applied in immunoinformatic analyses of many other complex pathogen genomes.

## Introduction

Cell-mediated immunity plays a central role in protection of the host against pathogens. The major key players of the cellular arm of the adaptive immune response are CD4+ and CD8+ T-cells, which recognize pathogenic determinants presented in the context of MHC molecules on professional antigen presenting cells (APCs). The contribution of the CD8 response, mediated by cytotoxic T-cells (CTLs), to protection in the case of intracellular pathogens, is well documented [1]–[4]. CTL epitopes are generated from proteins which are degraded by the proteasome, and are subsequently transported into the endoplasmatic reticulum (ER) by transporters associated with antigen processing (TAP), where they are subjected to further trimming. The binding of the processed peptides (8–11 amino acid in length) to the cleft of the various MHC-I alleles, is based on sequence features embedded in the peptide sequence and more specifically in anchor residues [5]. The identification of the presented MHC-peptide complex by the T-cell receptor triggers a whole cascade of cellular responses including cell proliferation and secretion of cytokines (such as IFNγ and IL-2).

*F. tularensis*, a relatively small Gram negative, facultative intracellular bacterium, is the etiological agent causing tularemia. The bacterium can infect many animal species, including humans. The severity of the disease depends on the strain and the route of infection. Several subspecies are recognized, namely *F. tularensis* tularensis (also referred to as type A strains), *F. tularensis* holarctica (type B strains), *F. tularensis* novicida and *F. tularensis* mediasiatica, where the two former are documented as human pathogens. Following inhalation of type A strains a respiratory disease may develop, which can lead to 30–60% mortality if left untreated [6]–[8]. Owing to the low respiratory lethal dose of the type A *F. tularensis* isolates and ease of aerosol release, these isolates were classified as Category A biothreat agents. These facts motivated in recent years elaborate research efforts aiming at establishing genetic tools, identification of virulence-related traits and development of novel countermeasures [9], [10]. Killed vaccines are highly reactogenic and poorly immunogenic, whereas the live attenuated LVS vaccine strain (a poorly characterized derivative of a type B strain) confers partial protection and suffers from side effects. In the absence of a licensed vaccine, major efforts are dedicated to the development of a safe and efficacious vaccine [11]–[17]. Genomic and proteomic identification of novel targets for attenuation and/or design of subunit vaccines are currently being pursued by various approaches. The methodologies underlying this large-scale, rational identification of novel vaccine targets rely on the availability of the complete genomic sequence of various pathogens. Such a “reverse vaccinology” approach was first applied for *Neisseria meningitidis* [18] and since then for several other pathogens [10], [19], [20]–[27]. The vital role of cellular immunity in defense against pathogens resulted in incorporation of the cellular immunity aspects into reverse vaccinology, sometimes referred to as “reverse immunology”. In parallel, a line of state-of-the-art computational tools were developed in recent years ([28]–[30] and references therein), which produce relatively accurate binding predictions to many MHC alleles (in particular for MHC class I), as well as prediction of proteasomal cleavage [31], [32] and TAP-related transport [33]. These methods were validated and proved to be highly reliable [34]–[38] and therefore may allow to decipher the potential repertoire of T-cell epitopes for a given pathogen.

In this study we describe a global immunoinformatic screen conducted on the genome sequence of *F. tularensis* LVS strain, in order to identify putative CTL epitopes and to evaluate their ability to elicit a T-cell response. In spite of the well acknowledged and documented contribution of CTLs to protection, the information on *F. tularensis* existing CTL epitopes which have been verified experimentally is very limited. Here we describe a strategy for whole genome down selection of candidates, based on mapping of clusters (“hotspots”) of putative MHC binders. This whole genome analysis approach was experimentally evaluated on a total of 1740 putative epitopes, of which 1240 were cluster-based selected peptides (subset I) and 500 were randomly selected putative, predicted MHC binders (subset II). The peptides were synthesized and tested for their ability to induce a CTL response. The results unequivocally demonstrate the strength of the developed strategy for enrichment of possible responders among the *in silico* candidates mapped from the complete theoretical bacterial proteome.

## Results

### Immunoinformatic identification of MHC class I putative binders

**Figure 1:**
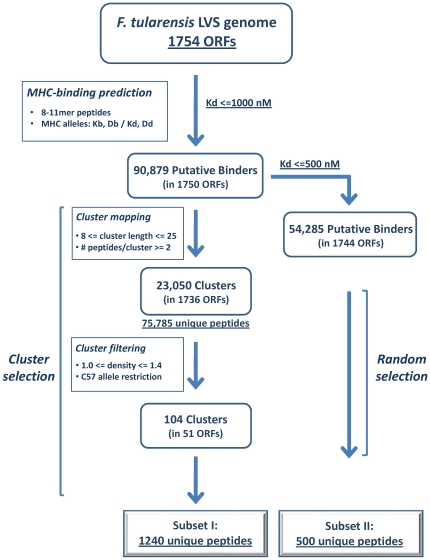
Flowchart of the whole-genome immunoinformatic analyses. A schematic representation of the reductive strategy conducted for down selection of putative MHC binders from the *F. tularensis* LVS genome and of the cluster based (Subset I) and the randomly chosen (Subset II) peptide selection for experimental evaluation.

The purpose of the study was to develop a strategy which will allow for identification of a significant fraction of the effective T-cell epitope repertoire, in a complete bacterial proteome. The analysis concentrated on prediction of peptide capability to bind MHC class I molecules, an event considered as the most selective in the cascade of events leading to epitope presentation on the surface of APCs (the analysis did not include the two other predictable pathway events, namely the proteasomal cleavage step and TAP binding and transport). The 1754 open reading frame (ORF) product sequences of *F. tularensis* can generate over 10^7^ possible peptides, 8, 9, 10 and 11-amino acid long. The first step in the analysis (Figure 1) mapped the putative MHC class I binders to the various mouse alleles, with predicted binding affinity equal or higher than 1000 nM. This analysis yielded a pool of 90,879 possible binders (encompassing 79,379 unique sequences) which originate from almost every ORF product in the genome (1750 out of 1754). This is still a very large library of possible binders to be handled experimentally, requiring the development and application of a rational strategy for a more rigorous down selection of candidates towards generation of a subset of peptides for further experimental evaluation.

### Cluster mapping and the selection of putative binders for experimental evaluation

With the intention to focus on antigen regions with maximal potential to elicit a CTL response, we decided to search for clusters of predicted binders along the entire proteome. As an operational definition, we define a cluster as a succession of at least two consecutive, overlapping predicted binders, with a maximal length of 25 amino acids. Accordingly, the minimal length of a cluster can be 8 amino acids if it contains a 8mer sequence recognizing two different H2 mouse alleles. Using these boundaries, a total number of 23,050 clusters could be identified. These contained 86,960 predicted binders, encompassing 75,785 unique sequences originating from 1736 proteins (Figure 1). Keeping in mind that we started with 90,879 putative binders, it is quite clear that the clustering procedure *per se* is insufficient to significantly reduce the number of peptides for experimental evaluation. A commonly used peptide ranking strategy applied for further down selection relies on binding affinities. However, we decided to undertake a different approach and to rank these clusters by the density of the peptides within each cluster (see Materials and Methods; for example, a density of 1.0 refers to 25 putative binders in a cluster of 25 amino acids). According to this approach, a versatile cutoff value for selection could be determined, depending on the size of the peptide library that one can afford to prepare for experimental evaluation. To test experimentally our approach, it was decided to generate a library of 1000–1500 peptides. Accordingly, we found that the top-ranking 104 clusters (having densities equal to or greater than 1.0) contain about 1400 peptides. In addition, we ensured that all 104 clusters include binders predicted to recognize the MHC alleles of the experimental animal model - C57BL (H2-Kb and H2-Db). We note that these 104 clusters represent a small fraction of all 23,050 mapped clusters. Overall, this reductive strategy (summarized in Figure 1) resulted in a total number of 1240 unique putative CTL epitopes and these peptides constituted the cluster-based subset of peptides (subset I) for further experimental evaluation (Table S1).

To test the validity of our approach, we also included a reference group of predicted MHC binders selected regardless of their location in clusters. This control group was composed of 500 potential CTL epitopes randomly selected out of the whole repertoire of predicted binders (Figure 1, subset II), and encompassing 402 proteins (Table S2). Rather than using the 1000 nM cutoff implemented for subset I, we employed a more stringent predicted binding affinity cutoff of 500 nM, to increase the chance of obtaining positive responders in this randomly selected subset. Aside from this restriction, no other restriction as inclusion criteria was imposed on the random set. The average density of clusters in the 402 proteins (0.36) is comparable to the average density of clusters in the whole genome (0.38). In addition, as one could expect from a non-biased set, the fraction of binders restricted to the mouse H2-Kb and H2-Db alleles in the random set is similar to that in the cluster-based peptide library (77.4% and 82%, respectively; Table S1 and Table S2). The random set of peptides constitutes therefore a “naïve” and suitable control set to evaluate the potential of the cluster-based approach for selection of putative CTL epitopes. Altogether we synthesized 1740 putative CTL epitopes for further experimental evaluation.

### Antigenicity of the selected peptides

**Figure 2:**
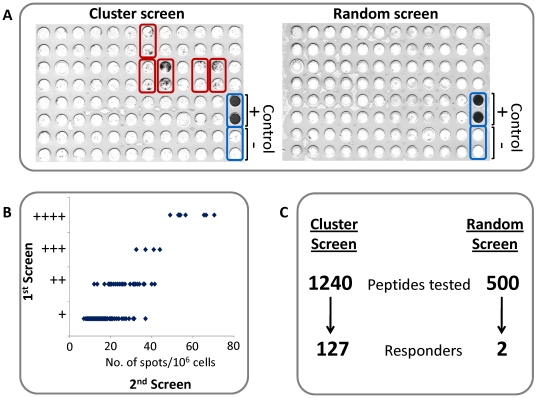
IFNγ EliSpot assay and results of the experimental screens. Sample EliSpot plates from the cluster screen of the 1240 cluster-derived peptides (left plate) and from the random screen of the 500 randomly selected MHC binders. Each peptide was tested in duplicates; positive responders are highlighted in red box, while positive and negative control wells are marked by a blue box. (B) Distribution of peptides shown to elicit IFNγ production, according to the extent of their response in the 1^st^ (“rough”) and the 2^nd^ (qualitative) screens. (C) Summary of the total numbers of tested and responding peptides, in each of the subsets evaluated. The 1240 tested peptides originate from the 104 top-ranking clusters (see Figure 1), and the 127 responder peptides were found to be located in 86 of these 104 clusters.

All 1740 potential MHC binders were tested for their ability to stimulate *F. tularensis* specific, K^b^ and D^b^-restricted T-cell response. To this end, groups of C57BL/6 mice were intranasally immunized with sub-lethal dose of live LVS, splenocytes from these mice were co-incubated with the individual peptides, and the extent of stimulation of IFNγ production was evaluated in an EliSpot assay (see Materials & Methods). Two consecutive screens were performed to identify potential responders: A “rough” screen followed by a second screen, which was more quantitative (Figure 2). All peptides which were considered as positive responders in the first screen were further confirmed as positive also in the second screen. Furthermore, the strong responders in the first screen ((+++) or (++++)), also exhibited the greatest extent of response in the second screen (30–80 spots/well; Figure 2). A total of 127 out of the 1240 peptides (10.7%) chosen by the cluster approach (subset I) were found to stimulate IFNγ production from splenocytes of LVS-immunized mice (Figure 2 and Table S1). In marked contrast to these results, of the randomly selected 500 potential CTL epitopes (control subset II), only 2 peptides (0.4%) were found to induce production of IFNγ (Figure 2 and Table S2).

### Distribution of the parental antigens according to functional categories

**Figure 3:**
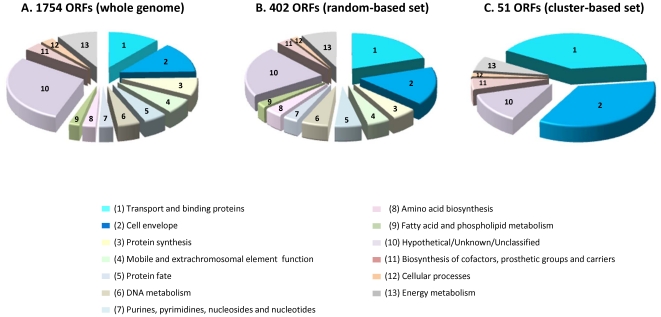
Distribution of *F. tularensis* proteins according to various functional categories. The classification of the *F. tularensis* LVS ORF sequences into the different functional categories was adapted from the CMR database (http://cmr.jcvi.org/tigr-scripts/CMR/CmrHomePage.cgi), with minor modifications. The population of the proteins in the different categories is presented for the whole genome 1740 ORF products (A), the 402 ORF products containing the 500 randomly-selected putative MHC binders (B) and the 51 ORF products containing all the 1240 tested set of MHC binders (C). Only functional categories with sufficiently high frequency in the whole genome (over 2%) were included in the chart.

As described earlier, the mapped clusters covered essentially the entire genome (1736 out of 1754 ORF products). Yet, the top-ranking 104 clusters selected for analysis of 1240 peptides represent only 51 ORF products. It was therefore interesting to determine whether this small number of ORF products populates functional categories enriched (or under-represented) in comparison to their fraction in the 402 proteins of the random-based set and to their fraction in the 1754 proteins of the whole genome. The classification of the proteins into functional categories was adapted from the CMR database (http://cmr.jcvi.org/tigr-scripts/CMR/CmrHomePage.cgi), and is presented in Figure 3. Most interestingly, a strong enrichment of two categories (Transport and binding proteins and Cell envelope) can be observed in the subset of 51 ORF products harboring the cluster-based selected peptides (Figure 3C), as compared to their frequency in the whole population (Figure 3A) and in the 402 ORF products harboring the random-based selected peptides (Figure 3B). These two categories are related to the cell membrane and presumably to membrane-associated proteins. None of the 51 proteins populate functional categories mostly associated with housekeeping genes, such as protein/amino acid biosynthesis, DNA and fatty acid metabolism. The percentage of ORF products populating the “unknown” functional category was found to be lower in the 51 proteins, while for the remaining categories, the frequency of proteins resembles their relevant frequency in the whole genome and in the random-based set. The 127 positive responders were found to be contained in 40 out of the 51 ORF products. These 40 ORF products exhibit a distribution of functional categories similar to that of the 51 proteins.

### Biochemical characteristics of the parental antigens

**Figure 4:**
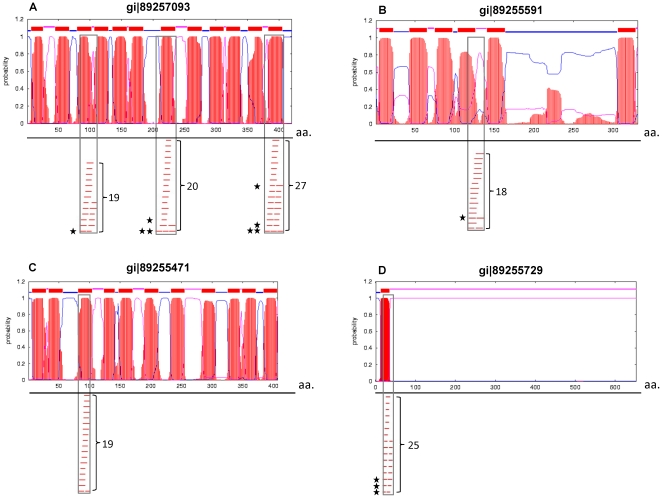
Alignment of “hotspots” of putative CTL epitopes with secondary structure domains. Sample plots of predicted helix-spanning regions of four selected proteins (graphical presentation of the output generated by TMHMM prediction), aligned with the highly-dense cluster regions of putative CTL peptides mapped in this study. Briefly, red segments represent the probability of having a helical region, while the thin blue and pink lines describe a possible topology of the membranal helices. In the lower part of every protein chart, a bar represents a predicted CTL binder. Co-localization of the putative MHC binders with the predicted helix or loop region in the protein is marked by a grey box. The total number of predicted CTL epitopes in the hotspot region is given on the rightside of the grey bar, and the stars on the leftside indicate responders. Note, that in example D, the predicted helix segment overlaps a signal peptide domain, which contains only one out of the three marked responders in the cluster.

The bias toward membrane-associated proteins as CTL peptide carriers in subset I, and the highly significant percent of responders within this subset as compared to the randomly selected peptide binders (subset II), prompted us to analyze the hydrophobicity/hydrophilicity character of the individual peptides (see Materials & Methods). The average hydropathy scores of the groups of peptides belonging to the cluster-based tested set (1240 peptides) and of the responders group (127 peptides) are 1.86 and 1.66, respectively. In both groups, the average hydropathy score values are much higher than the average values of 0.52–0.6 obtained for either peptides in subset II (the 500 peptides with IC_50_ values of 500 nM and below), or for the entire population of 90,879 peptides predicted as binders (IC_50_ values of 1000 nM and below), or the average values of the peptides considered essentially as non-binders (IC_50_>5000 nM). It therefore appears that peptides of subset I are quite hydrophobic, in accordance with the bias towards membrane-associated functional categories in the 51 proteins from which these peptides originated (Figure 3). Topological analysis of the parental antigen sequences for presence of transmembranal helices revealed that the 51 ORF products indeed show a strong bias towards integral membrane proteins (65%), compared to a much lower frequency of 30% in ORFs encompassing the peptides in the random set (subset II). Examination of these topological analyses provided some insights into the characteristics of the cluster regions within these proteins. In 70% of the cases, the peptides embedded in the cluster regions were found to co-localize with transmembranal helices and most of the remaining peptides overlapped with inter-connecting loops. These profiles are exemplified in Figure 4. We depict two prototypical membranal polypeptides containing clusters (Figure 4A and 4C), each cluster within these two polypeptides harbors about 20 predicted epitopes. For both polypeptides, the hotspots appear to overlap helical regions, but only in one protein, two clusters contain CTL responder peptides (Figure 4A). This example demonstrates that a highly dense cluster overlapping a helical region is not by itself sufficient to ensure the presence of a CTL responder. The example shown in Figure 4B demonstrates co-localization of a hotspot region in a loop between two helices (such loop region can also be seen in the N-terminal cluster of the protein presented in Figure 4A). Again, cases of clusters overlapping loop regions were found also in ORFs that did not contain any CTL responder (not shown). A total of 26 out of the 51 ORFs containing the high density clusters possess a signal peptide domain. Yet, only in few of these proteins (4 ORFs) this signal peptide harbored a cluster of predicted MHC binders. Interestingly, in all these 4 cases, the clusters contained at least one CTL responder peptide (cf. Figure 4D).

## Discussion

Unraveling the immune response elicited by a pathogen is of special significance for deciphering some of the organism routes of infection and mechanisms of pathogenesis as well as for vaccine development. Several studies relating to *in silico* mapping of T-cell determinants have been described, however these were mainly reported for individual viral or bacterial proteins, as well as tumor antigens. More extensive, genome-based screens were conducted mostly for the relatively small viral genomes. In the case of complex pathogens, such as for *M. tuberculosis* and *F. tularensis* [39]–[41], the screens included epitopes from subsets of antigens preselected according to particular criteria (e.g. secreted antigens). In view of documented evidence on the involvement of a relatively large fraction of the genome in the cellular response, it was recently stated [42] that there is a need for an unbiased analysis of the whole genome to complement the gap of knowledge that exists even for extensively studied pathogens, such as *Mycobacterium tuberculosis* [43].

Our study focused on CTL epitope analysis of the intracellular *F. tularensis* bacterium and to the best of our knowledge, it represents the first example of a comprehensive whole genome, *unbiased* attempt to screen for potential CTL epitopes in a complex pathogen. The laborious and costly experimental approaches for evaluation of predicted MHC binders as T-cell epitopes require the design of a rational filtering strategy, in particular in case of a bacterial proteome for which a vast number of potential candidates is generated by the predictions. We show in this study that *a priori* mapping of immunological hotspots constitutes an efficient strategy to enrich the population of responders by evaluation of a rather small number of potential determinants.

It is well established that there exists an overall correlation between MHC binding affinity and immunogenicity, where peptides having an IC_50_ value of 50 nM and less are considered as strong binders and IC_50_ values above 1000 nM are only occasionally detected. Indeed, studies analyzing the relation between binding affinities and MHC binding of peptides revealed that the vast majority of immunogenic peptides have a binding affinity below 500 nM [44], [45], [46]. In the present study, the IC_50_ values assigned to the peptides are theoretical rather than actually measured, and the mouse alleles under consideration are overall less represented in the predictor training sets. We therefore decided to start with a relatively tolerant affinity cutoff of 1000 nM, and to subject the rather large pool of mapped peptides (a total of 90,879) to additional filtering steps (Figure 1). The approach we have undertaken is based on identification of “hotspot” regions, namely regions of high density of predicted MHC binders embedded in stretches of up to 25 amino acids. Since we limited our analysis to a relatively small library size of about 1500 peptides, the cutoff of top-ranking clusters was determined at a density of 1.0 and above. Accordingly, we selected a pool of 104 top-ranking clusters containing 1240 peptides. This library of peptides was tested for its ability to elicit a T-cell response in an IFNγ-EliSpot assay. It was found that the majority of the clusters contained one or more positive CTL epitopes and out of the 1240 tested peptides, 127 were detected as responders. All 127 epitopes identified in this study are novel *F. tularensis* CTL epitopes and none overlap any of the 200 experimentally verified *F. tularensis* T-cell epitopes compiled from various studies in the Immune Epitope Database and Analysis Resource (IEDB, www.immuneepitope.org [47]). Thus by our approach, we have enlarged the library of *F. tularensis* T-cell epitopes by over 50%.

**Figure 5:**
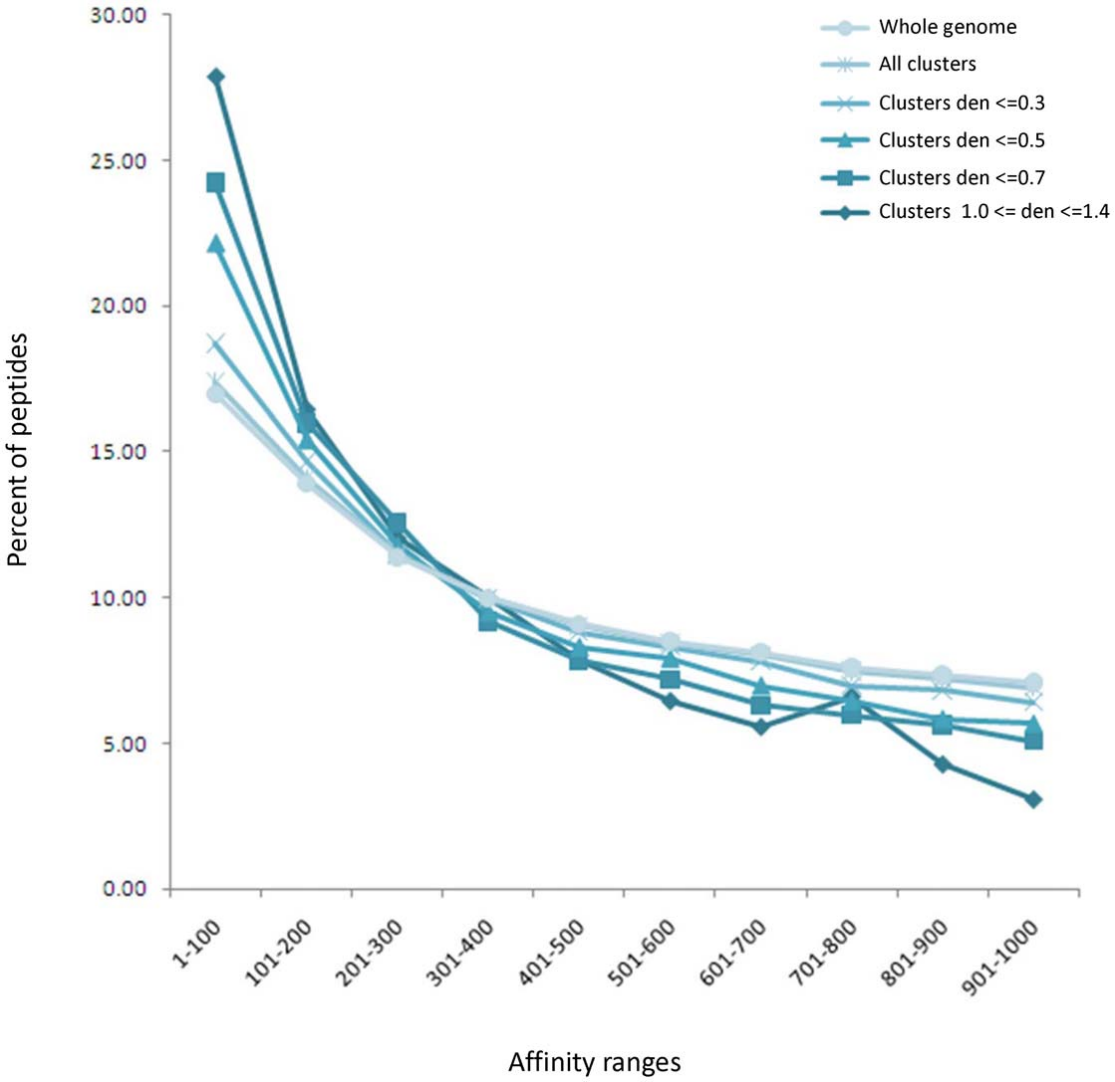
Distribution of peptide affinities in various pools of predicted MHC binders. The percent of peptides populating a range of affinities up to 1000 nM is indicated for the following pools: Whole genome (circle) −90,879 peptides; all mapped clusters (star) −75,785 peptides; clusters with densities up to 0.3 (x sign) −60,870 peptides, up to 0.5 (triangle) −26,699 peptides, up to 0.7 (square) −9,240 peptides, 1.0–1.4 (diamond) −1240 peptides.

Inspection of the subsets of mapped and evaluated peptides with respect to their affinities reveals that there is a clear enrichment of peptides with stronger predicted binding affinities (IC_50_ up to 300 nM) concomitant with the increase in the density of the parental cluster (Figure 5). While we find it difficult to provide an explanation for this observation it emphasizes the added value of focusing on relatively high cluster densities, and it may account, at least in part, for the observed enrichment of immunogenic peptides in these highly dense clusters. Yet, we note that due to our selection strategy, the peptides in the library and the responder peptides occupy a wide range of predicted affinities, and had we not included peptides with affinities of 500–1000 nM, we could have missed 28% of the responders.

Consequential to the selection strategy, a fraction of the selected peptides as well as the responders are “nested” peptides, and therefore might, theoretically, stimulate the same T-cell population. These nested versions of peptides depend on several events of degradation that might result in various peptide variations, including the variation that fits the requirement for protection of the epitope from peptidase degradation. Inspection of all the 66 sets of nested peptides (overall 166 peptides) among the 1240 cluster-based selected peptides evaluated in this study reveals, that no rule of thumb (e.g. predicted affinity or peptide length) exists to decide *a priori* which among these nested peptides will eventually elicit the cellular response (see Table S1). In over 50% of the cases the one peptide within the nested set which eventually elicited the response was not among the peptides exhibiting the highest predicted affinity (in ∼30% of the cases it had the lowest affinity; Table S1). Obviously, such nested peptides are scarce in the random set. Nevertheless, if one would consider only the “unique”, non-redundant responders (i.e. any two responder peptides overlapping by at least 7 residues are suspicious to stimulate a cross reactive response and therefore counted as one) then the number of responders should be 82 instead of 127 (Table S1). Even under such stringent assumptions the extent of response generated by the cluster approach is 6.6% which is still significantly high and about 17-fold higher than that found in the control random set. Deconvolution of the contribution of each of the nested peptides to stimulation of T-cell populations will be the subject of future studies.

The most striking observation is that the fraction of immunogenic hits obtained among the peptides selected by the cluster-based approach is 17–25 fold higher than in the case of responders in the *in silico* preselected peptides of the random set. The significance of this result is that the process of selection by highly dense clusters leads to a substantial enrichment of responders, without which almost 20,500 to 32,000 putative MHC binder peptides (instead of the 1240 peptides) would have had to be screened to identify a similar number of positive responders. It should be noted though, that the peptides included in the random set which were selected based on MHC binding alone, were not the top predicted binding peptides, but rather had overall comparable affinity to the cluster-based selected peptides. Therefore, a comparison of our screening strategy (selecting peptides from hotspots) vs. a conventional screening strategy (selecting the highest affinity peptides) remains to be done.

Previous reports on hotspot regions of CD8/CTL epitopes (as well as CD4/HTL epitopes [48], [49]) were documented in the experimental analysis of individual proteins [50]–[54]. The tendency of defined CTL epitopes to cluster was detected in the case of various HIV-1 proteins, and investigation of the amino acid sequences of some of these protein revealed that the clustered epitopes are concentrated in relatively conserved regions, an observation which was proposed by the authors to relate to viral variation and adaptation to the host. In addition, sites with large number of overlapping epitopes were found mostly in helical regions or in loops. Analysis of the CTL clustering in the HIV-1 Nef protein revealed that the sensitivity of hydrophobic regions to proteasomal processing is the major contributor to the epitope clustering in such regions [51]. A subsequent, comprehensive analysis of the HIV proteome corroborated previous findings on the correlation between epitope-rich regions and hydrophobicity, however the authors claimed that the predicted CTL epitopes in HIV-1 are randomly distributed [55]. Our comprehensive, genome-scale analysis demonstrates a clear enrichment (up to 70%) of membranal proteins containing highly dense regions of overlapping CTL epitopes. One could suggest that the enrichment of membranal proteins could be one of the reasons for the high success rate of positive hits obtained by our “hotspots” approach. It is important to note that in the control random set, the proportion of membranal proteins is about 30% (vs. 70% in the cluster-based set). This observation demonstrates that the membranal characteristic of the protein may at best account for 2.5-fold enrichment in responders and not to the 17–25 fold enrichment actually found in the cluster-based set. In addition, we have also shown that such highly dense preselected regions of putative epitopes frequently co-localize with hydrophobic regions, and most explicitly, with the helical segments of the proteins, as well as loops between helices (Figure 4).

To conclude, we have shown in this study that the strategy of *a priori* mapping immunological hotspots can be exploited to cover a relatively high percent of responders by testing a rather small number of potential CTL determinants. This approach led to a major increase (over 50%) in the number of documented T-cell epitopes of *F. tularensis*. A relatively high fraction of the determinants identified in this study are also identical to the orthologous sequences in the virulent *F. tularensis* tularensis Schu S4 strain, as could be anticipated from the extensive sequence identity between these two strains. Studies are now underway to evaluate the relevance of some of the responders, and specifically those which consistently exhibited a high level of stimulation of IFNγ production, for their contribution to protection against the virulent Schu S4 strain and for design of future vaccines. Finally, we believe that the application of the approach for enrichment of true positive responders, as demonstrated in this study, could be of general use for immunoinformatic analysis of other complex pathogens.

## Materials and Methods

### Ethics statement

This study was carried out in strict accordance with the recommendations in the Guide for the Care and Use of Laboratory Animals of the National Institute of Health. The protocol was approved by the Israel Institute for Biological Research Animal Care and Use Committee (Permit Number: IACUC-IIBR M-53-2009).

### Prediction of MHC class I putative binders

A total of 1754 *F. tularensis* ORF products (holarctica LVS strain, GenBank accession AM233362) were subjected to analysis for identification of putative CTL epitopes, by the MHC class I predictor NetMHC3.0 using artificial neural networks (ANN) [56]. Mouse MHC alleles were considered (H2-Kb, H2-Db, H2-Kd, H2-Dd). Predicted affinities (IC_50_, nM) for all 8, 9, 10, 11-mer peptides were computed. It should be mentioned that apparently, predictions of 8, 10 and 11-mer Db-restricted epitopes by the NetMHC3.0 approximation approach might be considered as less reliable (however in our hands the fraction of Db-restricted epitopes among the theoretical CTL epitopes and the responders was similar).

### Cluster mapping

A cluster of epitopes was defined as a polypeptide of up to 25 amino acids, containing at least 2 consecutive overlapping predicted binders. According to this designation, the dataset of all predicted binders was subjected to cluster mapping, using an *in house* developed software package for cluster mapping, data processing and visualization. The density of a given cluster was calculated as the number of predicted binders contained within the cluster divided by its length.

### Peptide synthesis

All peptides were synthesized by the 9-fluorenyl-methyloxycarbonyl (FMOC) chemistry and validated by mass spectrometry (Sigma, Israel). Peptides were adjusted to 500 µM stock solutions and stored at −20°C until use.

### Preparation of splenocytes

Animal procedures were approved by the Israel Institute for Biological Research Animal Care and Use Committee (Permit Number: IACUC-IIBR M-53-2009). Throughout the experiments, all efforts were made to minimize animal suffering. Groups of C57BL/6 mice were immunized with 10^2^ CFU LVS by the *i.n.* route after anesthesia with Ketamine and Xylazine. Six weeks later mice were euthanized, spleens removed and splenocytes were prepared using a gentle MACS C-tube (Milteny, Germany) according to the manufacturer's instructions. The freshly prepared splenocytes were suspended in RPMI-1640 supplemented with 10% heat inactivated fetal calf serum and 1 mM of Pen-Strep, non-essential amino acids, 2 mM L-glutamine and sodium pyruvate (all tissue culture solutions were obtained from Biological Industries, Bet Haemek, Israel).

### IFNγ EliSpot assay

A single-cell suspension of fresh splenocytes was seeded in EliSpot 96-well plates in complete RPMI medium containing 10 µM of each individual peptide or 10^7^ CFU/ml formalin inactivated LVS as a positive control. Each peptide sample was tested in duplicates. The frequency of epitope-specific T lymphocytes was determined using eBioscience IFNγ EliSpot kits with strict adherence to manufacturer's instructions. In the first screen (the “rough” screen) we used 10^6^ cells per well. A well was considered to be positive when it contained over 10 spots and had at least twice the number of spots counted in the negative control well. Throughout the screen, the background number of spots in negative control wells did not exceed five spots per well. The second screen (a more quantitative screen) included only the positive peptides from the first screen and contained less cells per well (5×10^5^ cells per well), which allowed a more accurate quantification of the results. In this second screen, the background number of spots in negative control wells was 0–1 and the number of spots among the positive peptide responders was at least 7. Throughout the experiment, the positive control (inactivated LVS) was confluent. Eventually, all of the responses that were considered positive according to the mentioned criteria were also positive by the two-tailed non-parametric Mann–Whitney *U*-test (at *p*<0.05).

### Transmembranal helices and signal peptide predictions

Analysis of membrane protein topology was conducted by the program TMHMM v2.0 [57] for prediction of transmembranal helices based on hidden Markov model. Analysis of proteins for presence of a signal peptide domain was performed by the Signalp 3.0 server, using the Neural networks (NN)-based method [58].

### Hydropathy score calculation

The hydropathicity of a peptide was calculated by an in-house script based on the GRAVY value (ExPASy Proteomics Server, http://expasy.org/tools/protparam-doc.html) defined as the sum of hydropathy scores of all its amino acids divided by the number of residues in the sequence. The scores are based on the amino acid indices derived by Kyte and Doolittle [59].

## Supporting Information

Table S1 List of 1240 peptides selected by the cluster-based approach. The affinity provided is the value predicted for a particular responder sequence by the NetMHC3.0 program. Cluster density is the number of predicted CTL epitopes in the responder parental cluster, divided by the parental cluster length. The gi number and annotation of the source protein are according to the *F. tularensis* holarctica LVS sequence deposited at the NCBI (GenBank accession AM233362); ^(a)^ Magnitude of T-cell response for each identified epitope is indicated as follows (expressed in SFC/million cells): L (low) - 7–19; M (medium) - 20–34; H (high) - 35 and above.
(PDF)

Table S2 List of 500 peptides selected by the random-based approach. The affinity provided is the value predicted for a particular responder sequence by the NetMHC3.0 program. Cluster density is the number of predicted CTL epitopes in the responder parental cluster, divided by the parental cluster length. The gi number and annotation of the source protein are according to the *F. tularensis* holarctica LVS sequence deposited at the NCBI (GenBank accession AM233362); ^(a)^ Magnitude of T-cell response for each identified epitope is indicated as follows (expressed in SFC/million cells): L (low) - 7–19; M (medium) - 20–34; H (high) - 35 and above.
(PDF)
