# Supplementary material for: Whole-Genome Immunoinformatic Analysis of F. tularensis: Predicted CTL Epitopes Clustered in Hotspots Are Prone to Elicit a T-Cell Response
Source: PLoS One. 2011 May 20;6(5):e20050. doi: 10.1371/journal.pone.0020050 (PMC3098878; doi:10.1371/journal.pone.0020050)
Supplement: Table S1 — List of 1240 peptides selected by the cluster-based approach. The affinity provided is the value predicted for a particular responder sequence by the NetMHC3.0 program. Cluster density is the number of predicted CTL epitopes in the responder parental cluster, divided by the parental cluster length. The gi number and annotation of the source protein are according to the F. tularensis holarctica LVS sequence deposited at the NCBI (GenBank accession AM233362); (a) Magnitude of T-cell response for each identified epitope is indicated as follows (expressed in SFC/million cells): L (low) - 7–19; M (medium) - 20–34; H (high) - 35 and above. (PDF) [file pone.0020050.s001.pdf]

Supplement Table 1: Compilation of data for 1240 cluster-based selected putative MHC binders (Subset I)

Sequence	Length	Predicted allele	Affinity (IC_{50}, nM)	Cluster density	Protein gi#	Protein annotation	Responder[a]
LGGIIFNIL	9	Kb	476	1.1	89255471	serine transporter	
LGGIIFNILYL	11	Kb	594	1.1			
GGIIFNILYL	10	Kb	648	1.1			
GIIFNILYL	9	Db	562	1.1			
IIFNILYL	8	Kb	309	1.1			
IIFNILYLFAI	11	Kb	388	1.1			
FNILYLFAI	9	Db	590	1.1			
FNILYLFAIL	10	Kb	391	1.1			
NILYLFAIL	9	Kb	292	1.1			
ILYLFAIL	8	Kb	11	1.1			
ILYLFAILP	9	Kb	326	1.1			
ILYLFAILPI	10	Kb	145	1.1			
ILYLFAILPIL	11	Kb	38	1.1			
LYLFAILPI	9	Kd	299	1.1			
LYLFAILPIL	10	Kb	449	1.1			
LYLFAILPILL	11	Kb	442	1.1			
YLFAILPIL	9	Kb	909	1.1			
YLFAILPILL	10	Kb	911	1.1			
FAILPILL	8	Db	135	1.1			
ITIILIFYYAL	11	Kb	38	1.4	89255508	hypothetical membrane protein	
TIILIFYYAL	10	Kb	63	1.4			
TIILIFYYALI	11	Kb	725	1.4			
IILIFYYA	8	Kb	641	1.4			
IILIFYYAL	9	Kb	13	1.4			
IILIFYYALI	10	Kb	129	1.4			
ILIFYYAL	8	Kb	45	1.4			
ILIFYYALI	9	Kb	296	1.4			
ILIFYYALINI	11	Kb	686	1.4			
LIFYYALI	8	Kb	80	1.4			
LIFYYALINI	10	Kb	422	1.4			
LIFYYALINIL	11	Kb	67	1.4			
IFYYALINI	9	Kb	445	1.4			
IFYYALINIL	10	Kb	46	1.4			
IFYYALINILL	11	Kb	107	1.4			
FYYALINI	8	Kd	110	1.4			
FYYALINIL	9	Kd	72	1.4			
FYYALINILL	10	Kd	253	1.4			
YYALINIL	8	Kd	266	1.4			
YALINILL	8	Db	41	1.4			
SKIFSYHYI	9	Db	391	1.0	89255524	B-type cytochrome	
KIFSYHYI	8	Kb	144	1.0			
FSYHYIFA	8	Kb	239	1.0			
FSYHYIFASV	10	Kb	125	1.0			
FSYHYIFASVL	11	Kb	45	1.0			
SYHYIFASV	9	Kd	90	1.0			
SYHYIFASVL	10	Kd	42	1.0			
SYHYIFASVLL	11	Kd	370	1.0			
YHYIFASV	8	Kb	11	1.0			
YHYIFASVL	9	Kb	244	1.0			
YHYIFASVLL	10	Kb	319	1.0			
HYIFASVL	8	Kd	36	1.0			
HYIFASVLL	9	Kd	347	1.0			
HYIFASVLLVL	11	Kd	208	1.0			
ASVLLVLVVL	10	Kb	383	1.0			
SVLLVLVVL	9	Kb	269	1.0			
INSYIINYKLL	11	Kb	328	1.0	89255563	hypothetical protein	
NSYIINYKL	9	Kb	667	1.0			
NSYIINYKLL	10	Kb	162	1.0			
SYIINYKL	8	Kd	297	1.0			
SYIINYKLL	9	Kb	258	1.0			
SYIINYKLLNI	11	Kd	421	1.0			L
YIINYKLL	8	Kb	41	1.0			
INYKLLNI	8	Kb	70	1.0			
INYKLLNINL	10	Kb	41	1.0			
INYKLLNINLI	11	Kb	94	1.0			
YKLLNINL	8	Db	669	1.0			L
YKLLNINLI	9	Db	187	1.0			M
LNINLINTQL	10	Kb	386	1.0			
INLINTQL	8	Kb	903	1.0			
NKYLGVPIFLL	11	Kb	912	1.1	89255570	ferrous iron transport protein	
KYLGVPIFL	9	Kd	24	1.1			
KYLGVPIFLL	10	Kd	139	1.1			
KYLGVPIFLLM	11	Kd	506	1.1			
LGVPIFLLMM	10	Kb	741	1.1			
GVPIFLLMM	9	Kb	877	1.1			
GVPIFLLMMYL	11	Kb	528	1.1			
VPIFLLMMYL	10	Kb	355	1.1			
VPIFLLMMYLM	11	Kb	775	1.1			
PIFLLMMYL	9	Kb	451	1.1			
PIFLLMMYLM	10	Kb	795	1.1			
IFLLMMYLM	9	Kb	627	1.1			L
IFLLMMYLMFL	11	Kb	542	1.1			
FLLMMYLM	8	Db	327	1.1			
FLLMMYLMFL	10	Db	152	1.1			
LLMMYLMFL	9	Kb	578	1.1			
LMMYLMFLFSI	11	Kb	260	1.1			
MMYLMFLFSI	10	Kb	54	1.1			
MYLMFLFSI	9	Kd	89	1.1			
MYLMFLFSITL	11	Kb	88	1.1			
YLMFLFSITL	10	Kb	519	1.1			
LMFLFSITL	9	Kb	20	1.1			
ISSSYQNV	8	Kb	26	1.0	89255591	phosphate transport protein	
ISSSYQNVSYM	11	Kb	161	1.0			

Sequence	Length	Predicted allele	Affinity (IC_{50}, nM)	Cluster density	Protein gi#	Protein annotation	Responder[a]
SSSYQNVSY	9	Dd	785	1.0			
SSSYQNVSYM	10	Kb	287	1.0			
SSYQNVSYM	9	Db	6	1.0			L
SSYQNVSYMTV	11	Db	30	1.0			
SYQNVSYM	8	Kd	116	1.0			
SYQNVSYMTV	10	Kd	175	1.0			
SYQNVSYMTVV	11	Kd	62	1.0			
QNVSYMTV	8	Kb	582	1.0			
VSYMTVVNKV	10	Kb	170	1.0			
VSYMTVVNKVL	11	Kb	47	1.0			
SYMTVVNKV	9	Kd	34	1.0			
SYMTVVNKVL	10	Kd	20	1.0			
SYMTVVNKVLI	11	Kd	11	1.0			
MTVVNKVLI	9	Db	202	1.0			
MTVVNKVLIPM	11	Db	180	1.0			
TVVNKVLIPM	10	Kb	338	1.0			
SSFMYLHF	8	Kb	29	1.0	89255714	hypothetical protein	
SSFMYLHFNYI	11	Kb	45	1.0			
SFMYLHFNYI	10	Kd	928	1.0			
FMYLHFNYI	9	Db	17	1.0			
FMYLHFNYIL	10	Kb	53	1.0			
FMYLHFNYILI	11	Db	15	1.0			
MYLHFNYI	8	Kd	207	1.0			
YLHFNYIL	8	Db	233	1.0			
YLHFNYILI	9	Db	15	1.0			
YLHFNYILII	10	Db	167	1.0			
LHFNYILI	8	Kb	552	1.0			
LHFNYILIIAL	11	Kb	97	1.0			
FNYILIIAL	9	Kb	54	1.0			
NYILIIAL	8	Kd	298	1.0			
IALTFCAI	8	Kb	397	1.0			
IALTFCAISTI	11	Kb	460	1.0			
LTFCAISTI	9	Kb	548	1.0			
TFCAISTI	8	Kd	201	1.0			
RVLNLLFYL	9	Kb	552	1.0	89255715	aromatic amino acid transporter of the HAAAP family	M
RVLNLLFYLL	10	Kb	626	1.0			
RVLNLLFYLLL	11	Kb	444	1.0			
LNLLFYLL	8	Kb	24	1.0			
LNLLFYLLL	9	Kb	58	1.0			
LNLLFYLLLL	10	Kb	108	1.0			
LLFYLLLLYSL	11	Kb	82	1.0			
LFYLLLLYSL	10	Kb	70	1.0			
LFYLLLLYSLL	11	Kb	111	1.0			
FYLLLLYSL	9	Kd	15	1.0			
FYLLLLYSLL	10	Kd	21	1.0			
YLLLLYSLL	9	Kb	444	1.0			
LLLLYSLL	8	Kb	304	1.0			
LLYSLLTAYI	10	Kb	416	1.0			
LYSLLTAYI	9	Kd	498	1.0			
YSLLTAYI	8	Db	19	1.0			
YSLLTAYIF	9	Db	728	1.0			L
YSLLTAYIFM	10	Db	6	1.0			H
SLLTAYIFM	9	Db	49	1.0			
LTAYIFMGGQL	11	Kb	729	1.0			L
TAYIFMGGQL	10	Kb	31	1.0			
AYIFMGGQL	9	Kd	14	1.0			
MALIFSFV	8	Kb	326	1.0	89255729	hypothetical membrane protein	
MALIFSFVIM	10	Kb	142	1.0			
MALIFSFVIMV	11	Kb	943	1.0			
ALIFSFVIM	9	Kb	771	1.0			
LIFSFVIM	8	Kb	140	1.0			M
LIFSFVIMV	9	Kb	525	1.0			
LIFSFVIMVM	10	Kb	135	1.0			
LIFSFVIMVML	11	Kb	102	1.0			
FSFVIMVM	8	Db	984	1.0			
FSFVIMVML	9	Kb	328	1.0			
SFVIMVMLTGL	11	Kd	135	1.0			
VIMVMLTGL	9	Kb	464	1.0			L
VIMVMLTGLL	10	Kb	803	1.0			
IMVMLTGL	8	Kb	550	1.0			
IMVMLTGLL	9	Kb	314	1.0			
IMVMLTGLLYI	11	Db	151	1.0			
VMLTGLLYI	9	Db	21	1.0			
TGLLYIFKI	9	Kb	510	1.0			
TGLLYIFKIGL	11	Kb	41	1.0			
GLLYIFKIGL	10	Kb	988	1.0			M
LLYIFKIGL	9	Kb	45	1.0			
LLYIFKIGLL	10	Kb	110	1.0			
LYIFKIGL	8	Kd	269	1.0			
LYIFKIGLL	9	Kd	632	1.0			
YIFKIGLL	8	Kb	703	1.0			
LTLVAFICVL	10	Kb	305	1.2	89255776	4-hydroxybenzoate octaprenyltransferase	
TLVAFICVL	9	Kb	383	1.2			
VAFICVLFL	9	Kb	296	1.0			
VAFICVLFLNL	11	Kb	126	1.0			
ICVLFLNL	8	Kb	101	1.0			
ICVLFLNLYTI	11	Db	553	1.0			
CVLFLNLYTI	10	Db	466	1.0			
CVLFLNLYTIL	11	Kb	114	1.0			
VLFLNLYTI	9	Db	31	1.0			
VLFLNLYTIL	10	Kb	122	1.0			L
VLFLNLYTILL	11	Kb	106	1.0			
LFLNLYTIL	9	Kd	368	1.0			
LFLNLYTILL	10	Kb	366	1.0			
LYTILLSFV	9	Kd	250	1.0			
LYTILLSFVAL	11	Kd	135	1.0			
YTILLSFVAL	10	Kb	406	1.0			

Sequence	Length	Predicted allele	Affinity (IC_{50}, nM)	Cluster density	Protein gi#	Protein annotation	Responder[(a)]
TILLSFVAL	9	Kb	59	1.0			
TILLSFVALFL	11	Kb	584	1.0			
ILLSFVAL	8	Kb	118	1.0			
ILLSFVALFL	10	Kb	517	1.0			
LSFVALFL	8	Kb	545	1.0			
LSFVALFLAI	10	Kb	645	1.0			
LSFVALFLAIL	11	Kb	149	1.0			
FVALFLAIL	9	Kb	567	1.0			
VALFLAIL	8	Kb	494	1.0			
VALFLAILYP	10	Kb	684	1.0			
NKVFRYILL	9	Kb	453	1.0			
KVFRYILL	8	Kb	22	1.0			
FRYILLFNFL	10	Kb	182	1.0			
RYILLFNFL	9	Kd	17	1.0			
RYILLFNFLSL	11	Kd	48	1.0			
YILLFNFL	8	Kb	197	1.0			
YILLFNFLSL	10	Kb	228	1.0			
YILLFNFLSLL	11	Kb	445	1.0			
ILLFNFLSL	9	Kb	47	1.0			
ILLFNFLSLL	10	Kb	111	1.0			
ILLFNFLSLLL	11	Kb	284	1.0			
LLFNFLSL	8	Kb	329	1.0			
LLFNFLSLL	9	Kb	140	1.0			
LLFNFLSLLL	10	Kb	302	1.0			
LLFNFLSLLLL	11	Kb	280	1.0			
LSLLLLII	8	Db	993	1.2			
LSLLLLIIL	9	Kb	79	1.2			
LSLLLLIILGI	11	Kb	812	1.1			
PTMKKYYYNTL	11	Kb	102	1.1	89255853	major facilitator superfamily (MFS) transport protein	
TMKKYYYNTL	10	Kb	36	1.1			
MKKYYYNTL	9	Kb	45	1.1			
KKYYYNTL	8	Kb	218	1.1			
KKYYYNTLIIL	11	Kb	163	1.1			
KYYYNTLI	8	Kd	90	1.1			
KYYYNTLII	9	Db	301	1.1			
KYYYNTLIIL	10	Kb	208	1.1			
KYYYNTLIILL	11	Kb	156	1.1			
YYYNTLII	8	Kd	696	1.1			
YYYNTLIIL	9	Kb	319	1.1			
YYYNTLIILL	10	Kb	243	1.1			
YYYNTLIILLL	11	Kb	454	1.1			
YYNTLIIL	8	Kd	86	1.1			
YYNTLIILL	9	Kd	214	1.1			
YYNTLIILLL	10	Kd	479	1.1			
LIILLLFFL	9	Kb	806	1.1			
LIILLLFFLAL	11	Kb	180	1.1			
IILLLFFLAL	10	Kb	41	1.1			
IILLLFFLALL	11	Kb	65	1.1			
ILLLFFLAL	9	Kb	42	1.1			
ILLLFFLALL	10	Kb	66	1.1			
LLLFFLALL	9	Kb	116	1.1			
FFLALLNYV	9	Kd	189	1.1			
GGFVIGFYSIL	11	Kb	877	1.0			
FVIGFYSI	8	Kb	243	1.0			
FVIGFYSIL	9	Kb	307	1.0			
FVIGFYSILL	10	Kb	431	1.0			
VIGFYSIL	8	Kb	755	1.0			
VIGFYSILL	9	Kb	598	1.0			
IGFYSILL	8	Kb	599	1.0			L
IGFYSILLTRI	11	Kb	191	1.0			
GFYSILLTRI	10	Kd	659	1.0			
FYSILLTRI	9	Kd	14	1.0			
FYSILLTRIL	10	Kd	726	1.0			M
FYSILLTRILL	11	Kd	284	1.0			
YSILLTRI	8	Db	402	1.0			
YSILLTRIL	9	Kb	816	1.0			
YSILLTRILL	10	Db	229	1.0			
SILLTRILL	9	Kb	215	1.0			
SILLTRILLGL	11	Kb	364	1.0			
ILLTRILLGL	10	Kb	948	1.0			
LAFLFFFFFL	10	Kb	27	1.1	89255996	Membrane protein/O-antigen protein	L
AFLFFFFFL	9	Kb	214	1.1			
AFLFFFFFLSM	11	Kb	81	1.1			
FLFFFFFLSM	10	Kb	69	1.1			
FLFFFFFLSMI	11	Kb	595	1.1			
LFFFFFLSM	9	Kb	44	1.1			
LFFFFFLSMI	10	Kb	461	1.1			
FFFFFLSMI	9	Kd	712	1.1			
FFFFFLSMIYL	11	Kb	253	1.1			
FFFFLSMIYL	10	Kb	659	1.1			
FFFLSMIYL	9	Db	272	1.1			
FFLSMIYL	8	Kd	249	1.1			
FFLSMIYLI	9	Kd	814	1.1			
LSMIYLIIVQI	11	Kb	562	1.1			
SMIYLIIVQI	10	Kb	330	1.1			
SMIYLIIVQII	11	Kb	812	1.1			
MIYLIIVQI	9	Kb	360	1.1			
MIYLIIVQII	10	Kb	881	1.1			
MIYLIIVQIIL	11	Kb	109	1.1			
IYLIIVQI	8	Kd	279	1.1			
IYLIIVQIIL	10	Kb	689	1.1			
IYLIIVQIILL	11	Kb	372	1.1			L
LIIVQIILL	9	Kb	910	1.1			
VQIILLDAASL	11	Kb	94	1.1			
QIILLDAASL	10	Kb	924	1.1			
IILLDAASL	9	Kb	118	1.1			
IGYIKYLYYYG	11	Kb	398	1.0			M

Sequence	Length	Predicted allele	Affinity (IC_{50}, nM)	Cluster density	Protein gi#	Protein annotation	Responder[(a)]
GYIKYLYYYGI	11	Kd	547	1.1			
IKYLYYYGI	9	Kb	48	1.1			
IKYLYYYGII	10	Kb	97	1.1			
KYLYYYGI	8	Kd	430	1.1			
KYLYYYGII	9	Kd	303	1.1			
YLYYYGIIFSM	11	Kb	124	1.1			
LYYYGIIFSM	10	Kb	45	1.1			
YYYGIIFSM	9	Kb	522	1.1			
YGIIFSMFFYI	11	Db	383	1.1			
IIFSMFFYI	9	Kb	189	1.1			
IIFSMFFYIL	10	Kb	50	1.1			
IIFSMFFYILI	11	Kb	135	1.1			
IFSMFFYIL	9	Kb	821	1.0			M
FSMFFYIL	8	Kb	30	1.1			
FSMFFYILI	9	Db	91	1.1			
FSMFFYILII	10	Db	163	1.1			
SMFFYILI	8	Kb	301	1.1			
SMFFYILII	9	Kb	269	1.1			
SMFFYILIIFL	11	Kb	33	1.1			
MFFYILIIFL	10	Kb	410	1.1			
FFYILIIFL	9	Kb	505	1.1			
FYILIIFL	8	Kd	58	1.1			
YILIIFLYFEM	11	Kb	957	1.1			
ILIIFLYFEM	10	Kb	297	1.0			L
LIIFLYFEM	9	Kb	198	1.1			
IIFLYFEM	8	Kb	78	1.0			M
LYFEMRKCFI	10	Kd	21	1.1			
LFICYVSTNI	10	Kd	822	1.0	89256001	O-antigen flippase	
ICYVSTNIM	9	Kb	176	1.0			H
CYVSTNIM	8	Kd	146	1.0			M
YVSTNIMYYFV	11	Db	339	1.0			
STNIMYYFV	9	Db	988	1.0			
TNIMYYFV	8	Kb	44	1.0			
NIMYYFVYQTI	11	Kb	830	1.0			
IMYYFVYQT	9	Kb	702	1.0			
IMYYFVYQTI	10	Kb	15	1.0			
IMYYFVYQTII	11	Kb	48	1.0			
MYYFVYQT	8	Kb	812	1.0			
MYYFVYQTI	9	Kb	177	1.0			
MYYFVYQTII	10	Kd	132	1.0			
YYFVYQTI	8	Kd	82	1.0			
YYFVYQTII	9	Kd	23	1.0			
YYFVYQTIIAI	11	Kd	85	1.0			
FVYQTIIAI	9	Kb	135	1.0			
FVYQTIIAIL	10	Kb	65	1.0			
VYQTIIAI	8	Kd	98	1.0			
VYQTIIAIL	9	Kd	409	1.0			
VYQTIIAILYL	11	Kd	555	1.0			
IAILYLVCI	9	Db	153	1.1			
IAILYLVCIAI	11	Db	260	1.0			
ILYLVCIAI	9	Kb	790	1.1			
LYLVCIAI	8	Kd	99	1.1			
AIIFLKEKVSL	11	Kb	193	1.0	89256054	conservered hypothetical membrane protein	
IIFLKEKVSL	10	Kb	67	1.0			
IFLKEKVSL	9	Kb	518	1.0			
VSLKYYFV	8	Kb	7	1.0			
VSLKYYFVL	9	Kb	19	1.0			
VSLKYYFVLL	10	Kb	14	1.0			L
VSLKYYFVLLL	11	Kb	28	1.0			
SLKYYFVLL	9	Kb	306	1.0			
SLKYYFVLLL	10	Kb	762	1.0			
LKYYFVLL	8	Kb	239	1.0			M
LKYYFVLLL	9	Kb	29	1.0			
KYYFVLLL	8	Kb	757	1.0			
KYYFVLLLAFL	11	Kd	167	1.0			
YYFVLLLAFL	10	Kd	165	1.0			
FVLLLAFL	8	Db	379	1.0			
FVLLLAFLGL	10	Kb	663	1.0			
FVLLLAFLGLL	11	Kb	719	1.0			
VLLLAFLGL	9	Kb	502	1.0			
VLLLAFLGLL	10	Kb	518	1.0			
VLLLAFLGLLL	11	Kb	966	1.0			
LLLAFLGL	8	Kb	865	1.0			
LLLAFLGLL	9	Kb	590	1.0			
LLLAFLGLLLL	11	Kb	844	1.0			
LAFLGLLLL	9	Kb	187	1.1			
LAFLGLLLLM	10	Kb	214	1.0			
LLLLMSQAGL	10	Kb	515	1.1			
LLLMSQAGL	9	Kb	727	1.1			
ITAFIFTSV	9	Kb	836	1.2	89256057	hypothetical protein	
ITAFIFTSVL	10	Kb	156	1.2			
TAFIFTSV	8	Kb	96	1.2			
TAFIFTSVL	9	Kb	27	1.2			
TSVLGYYYYV	10	Db	98	1.2			
TSVLGYYYYVM	11	Db	28	1.2			
SVLGYYYYV	9	Kb	736	1.2			
SVLGYYYYVM	10	Kb	97	1.2			
SVLGYYYYVMI	11	Kb	280	1.2			
VLGYYYYVM	9	Kb	305	1.2			
VLGYYYYVMI	10	Kb	916	1.2			
VLGYYYYVMIL	11	Kb	496	1.2			
LGYYYYVM	8	Kb	24	1.2			
LGYYYYVMI	9	Kb	14	1.2			
LGYYYYVMIL	10	Kb	9	1.2			
LGYYYYVMILA	11	Kb	307	1.2			
GYYYYVMI	8	Kb	838	1.2			
GYYYYVMIL	9	Kb	177	1.2			

Sequence	Length	Predicted allele	Affinity (IC_{50}, nM)	Cluster density	Protein gi#	Protein annotation	Responder[(a)]
YYYYVMIL	8	Kb	709	1.2			
YYYYVMILAV	10	Kd	967	1.2			
YYYVMILAV	9	Kd	701	1.2			
YYYVMILAVAI	11	Kd	98	1.2			
YYVMILAV	8	Kd	258	1.2			
YYVMILAVAI	10	Kd	25	1.2			
VAISVFITASI	11	Kb	259	1.0			
ISVFITASI	9	Kb	195	1.0			
SVFITASI	8	Kb	715	1.0			
QSALHFTLYL	10	Kb	668	1.0	89256070	polyamine transporter, subunit H, ABC transporter, membrane protein	L
SALHFTLYL	9	Kb	57	1.0			
LHFTLYLSNYI	11	Kb	907	1.0			
FTLYLSNYI	9	Kb	463	1.0			
FTLYLSNYIEL	11	Kb	103	1.0			
TLYLSNYIEL	10	Kb	139	1.0			
TLYLSNYIELI	11	Kb	956	1.0			
LYLSNYIEL	9	Db	25	1.0			
LYLSNYIELI	10	Kd	249	1.0			
SNYIELIHYNL	11	Kb	124	1.0			
NYIELIHYNL	10	Kd	941	1.0			
NYIELIHYNLI	11	Kd	783	1.0			
IELIHYNL	8	Kb	580	1.0			
IELIHYNLI	9	Kb	456	1.0			
IELIHYNLIFI	11	Db	849	1.0			
LIHYNLIFI	9	Db	57	1.0			
LIHYNLIFISL	11	Kb	80	1.0			M
IHYNLIFI	8	Kb	493	1.0			L
IHYNLIFISL	10	Kb	21	1.0			L
IHYNLIFISLL	11	Kb	47	1.0			
HYNLIFISL	9	Kd	34	1.1			
HYNLIFISLL	10	Kd	75	1.0			
YNLIFISL	8	Kb	40	1.1			
YNLIFISLL	9	Kb	247	1.1			
TITSMTNNRSL	11	Kb	837	1.0	89256087	hypothetical protein	
ITSMTNNRSL	10	Kb	825	1.0			
ITSMTNNRSLL	11	Kb	951	1.0			
TSMTNNRSL	9	Db	298	1.0			
TSMTNNRSLL	10	Db	184	1.0			
SMTNNRSLL	9	Kb	577	1.0			
RSLLKYFFV	9	Db	115	1.0			
RSLLKYFFVL	10	Db	84	1.0			
RSLLKYFFVLL	11	Kb	79	1.0			L
SLLKYFFVL	9	Kb	218	1.0			L
SLLKYFFVLL	10	Kb	96	1.0			
LLKYFFVLL	9	Kb	248	1.0			
LKYFFVLL	8	Kb	165	1.0			
LKYFFVLLI	9	Kb	318	1.0			
LKYFFVLLIL	10	Kb	93	1.0			
KYFFVLLI	8	Kd	478	1.0			
KYFFVLLIL	9	Kd	553	1.0			
KYFFVLLILFI	11	Kd	303	1.0			
FVLLILFI	8	Db	235	1.0			
ALIYMFFYFSI	11	Kb	339	1.0	89256094	hypothetical membrane protein	
LIYMFFYF	8	Kb	265	1.0			
LIYMFFYFSI	10	Kb	30	1.0			
LIYMFFYFSII	11	Kb	122	1.0			
IYMFFYFSI	9	Kb	86	1.0			
IYMFFYFSII	10	Kb	321	1.0			
YMFFYFSI	8	Kb	768	1.0			
YMFFYFSII	9	Kb	282	1.0			
FYFSIISYYI	10	Kd	23	1.0			
FSIISYYI	8	Db	10	1.0			
ISYYIYNY	8	Kb	549	1.0			
ISYYIYNYS	9	Kb	232	1.0			
ISYYIYNYSS	10	Kb	676	1.0			
ISYYIYNYSSK	11	Kb	656	1.0			
SYYIYNYS	8	Kb	766	1.0			
SYYIYNYSSKI	11	Kb	697	1.0			
YYIYNYSSKI	10	Kd	67	1.0			
YIYNYSSK	8	Kb	559	1.0			
IYNYSSKI	8	Kd	475	1.0			
NGLEFFYAYI	10	Kb	367	1.0	89256124	hypothetical membrane protein	
LEFFYAYI	8	Kb	420	1.0			
LEFFYAYIAFL	11	Kb	216	1.0			L
EFFYAYIAFL	10	Kb	724	1.0			
FFYAYIAF	8	Kb	675	1.0			
FFYAYIAFL	9	Kb	277	1.0			
FFYAYIAFLIL	11	Kb	563	1.0			
FYAYIAFL	8	Kd	391	1.0			
FYAYIAFLI	9	Kd	855	1.0			L
YAYIAFLI	8	Db	779	1.0			
YAYIAFLIL	9	Kb	180	1.0			
YAYIAFLILL	10	Kb	61	1.0			
AYIAFLIL	8	Kd	847	1.0			L
AYIAFLILL	9	Kb	244	1.0			
IAFLILLCYP	10	Kb	346	1.0			
IAFLILLCYPM	11	Kb	48	1.0			
AFLILLCYPM	10	Kb	885	1.0			M
FLILLCYPM	9	Db	148	1.0			H
LCYPMNIAAL	10	Kb	78	1.0			L
CYPMNIAAL	9	Kb	64	1.0			
CYPMNIAALYF	11	Dd	757	1.0			
IKFMLLVIYI	10	Kb	308	1.0	89256142	hypothetical membrane protein	
IKFMLLVIYII	11	Kb	821	1.0			
FMLLVIYI	8	Db	21	1.0			
FMLLVIYII	9	Db	73	1.0			
FMLLVIYIII	10	Db	37	1.0			

Sequence	Length	Predicted allele	Affinity (IC_{50}, nM)	Cluster density	Protein gi#	Protein annotation	Responder[(a)]
FMLLVIYIIIL	11	Db	75	1.0			
MLLVIYIIIL	10	Kb	949	1.0			
LLVIYIIIL	9	Kb	755	1.0			
LVIYIIILQYL	11	Kb	107	1.0			
VIYIIILQYL	10	Kb	39	1.0			
VIYIIILQYLL	11	Kb	69	1.0			
IYIIILQYL	9	Kd	105	1.0			
IYIIILQYLL	10	Kd	575	1.0			
IIILQYLL	8	Kb	402	1.0			
IIILQYLLSP	10	Kb	878	1.0			
IIILQYLLSPI	11	Kb	405	1.0			L
IILQYLLSP	9	Kb	291	1.0			
IILQYLLSPI	10	Kb	135	1.0			
IILQYLLSPIV	11	Kb	801	1.0			
LQYLLSPI	8	Kb	716	1.0			
LQYLLSPIV	9	Kb	599	1.0			
QYLLSPIV	8	Kd	823	1.0			
LSPIVHTSI	9	Kb	712	1.0			
NSISTVLYFI	10	Db	156	1.0	89256223	Sodium-dicarboxylate symporter family protein	
NSISTVLYFIL	11	Db	717	1.0			
ISTVLYFIL	9	Kb	561	1.0			L
STVLYFIL	8	Kb	164	1.0			
TVLYFILTCYI	11	Kb	172	1.0			
VLYFILTCYI	10	Kb	309	1.0			
LYFILTCYI	9	Kd	76	1.0			
LYFILTCYIAL	11	Kb	89	1.0			L
YFILTCYI	8	Kd	327	1.0			
FILTCYIAL	9	Kb	440	1.0			
ILTCYIAL	8	Kb	249	1.0			L
TCYIALIIVL	10	Kb	111	1.0			
TCYIALIIVLI	11	Kb	783	1.0			
CYIALIIV	8	Kd	759	1.0			
CYIALIIVL	9	Kd	260	1.0			
CYIALIIVLI	10	Kd	182	1.0			
IALIIVLIM	9	Kb	416	1.0			
LIIVLIMHITL	11	Kb	422	1.0			
IIVLIMHITL	10	Kb	220	1.0			
IIVLIMHITLL	11	Kb	451	1.0			
IVLIMHITL	9	Kb	40	1.0			
IVLIMHITLL	10	Kb	80	1.0			
VLIMHITLL	9	Kb	357	1.0			
VLIMHITLLVL	11	Kb	764	1.0			
IMHITLLVL	9	Db	136	1.0			
ISMTVYYL	8	Kb	32	1.0	89256227	hypothetical protein	L
ISMTVYYLVL	10	Db	162	1.0			L
SMTVYYLV	8	Kb	342	1.0			
SMTVYYLVL	9	Db	359	1.0			
MTVYYLVL	8	Kb	372	1.0			
MTVYYLVLNM	10	Kb	312	1.0			
TVYYLVLNM	9	Kb	44	1.0			
TVYYLVLNMHI	11	Kb	337	1.0			
VYYLVLNM	8	Kb	74	1.0			
VYYLVLNMHI	10	Kd	200	1.0			
YYLVLNMHI	9	Kd	29	1.0			
YLVLNMHI	8	Db	181	1.0			
LNMHIKNFIMM	11	Kb	568	1.0			L
NMHIKNFIMM	10	Db	460	1.0			
MHIKNFIMM	9	Kb	448	1.0			
ISFYGVLL	8	Kb	391	1.0	89256270	hypothetical membrane protein	L
ISFYGVLLI	9	Kb	371	1.0			
FYGVLLIFFI	10	Kd	295	1.0			L
YGVLLIFFI	9	Db	345	1.0			
VLLIFFIFAFI	11	Kb	824	1.0			
LLIFFIFAFIL	11	Kb	385	1.0			
LIFFIFAFI	9	Kb	603	1.0			
LIFFIFAFIL	10	Kb	155	1.0			
IFFIFAFI	8	Kb	532	1.0			
IFFIFAFIL	9	Kb	284	1.0			
FIFAFILF	8	Kb	633	1.0			
FIFAFILFYL	10	Kb	192	1.0			
FIFAFILFYLL	11	Kb	219	1.0			
FAFILFYL	8	Db	209	1.0			
FAFILFYLL	9	Kb	93	1.0			
FAFILFYLLV	10	Kb	775	1.0			
AFILFYLL	8	Kb	459	1.0			
AFILFYLLVNL	11	Kb	339	1.0			
FILFYLLV	8	Kb	620	1.0			
FILFYLLVNL	10	Kb	259	1.0			
ILFYLLVNL	9	Kb	63	1.0			L
LFYLLVNL	8	Kb	189	1.0			
FYLLVNLNV	9	Kd	307	1.0			
FYLLVNLNVF	10	Kd	819	1.0			
YLLVNLNVF	9	Db	376	1.0			
LLVNLNVFSSL	11	Kb	721	1.0			M
LVNLNVFSSL	10	Kb	675	1.0			L
VNLNVFSSL	9	Kb	57	1.0			
VNLNVFSSLNL	11	Kb	424	1.0			
NVFSSLNLVIL	11	Kb	591	1.0			
FSSLNLVI	8	Db	120	1.0			
FSSLNLVIL	9	Db	16	1.0			
FSSLNLVILPL	11	Db	96	1.0			L
SSLNLVIL	8	Kb	349	1.0			
SSLNLVILPL	10	Kb	57	1.0			
FALLIIFI	8	Db	25	1.0	89256312	Proton-dependent oligopeptide transport (POT) family protein	
FALLIIFITV	10	Db	246	1.0			
FALLIIFITVL	11	Db	55	1.0			
ALLIIFITVL	10	Kb	370	1.0			

Sequence	Length	Predicted allele	Affinity (IC_{50}, nM)	Cluster density	Protein gi#	Protein annotation	Responder[(a)]
ALLIIFITVLL	11	Kb	470	1.0			
LLIIFITVL	9	Kb	321	1.0			
LLIIFITVLL	10	Kb	399	1.0			
LLIIFITVLLL	11	Kb	826	1.0			
LIIFITVLL	9	Kb	305	1.0			
LIIFITVLLL	10	Kb	698	1.0			
IIFITVLL	8	Kb	166	1.0			
IIFITVLLL	9	Kb	241	1.0			
IFITVLLL	8	Kb	680	1.0			
ITVLLLFI	8	Db	912	1.0			
ITVLLLFIAL	10	Kb	65	1.0			
TVLLLFIAL	9	Kb	24	1.0			
VLLLFIAL	8	Kb	199	1.0			
LLLFIALQYPL	11	Kb	411	1.0			
LLFIALQYPL	10	Kb	238	1.0			
LFIALQYPL	9	Kd	592	1.0			M
LSMIAMTLL	9	Kb	440	1.0	89256315	hypothetical membrane protein	
LSMIAMTLLTM	11	Kb	336	1.0			H
SMIAMTLL	8	Db	487	1.0			
SMIAMTLLTM	10	Db	60	1.0			
IAMTLLTM	8	Db	93	1.0			H
IAMTLLTMFL	10	Db	189	1.0			
MTLLTMFL	8	Kb	794	1.0			
MTLLTMFLQYI	11	Kb	836	1.0			
LTMFLQYIGI	10	Kb	745	1.0			
TMFLQYIGI	9	Kb	75	1.0			L
TMFLQYIGIFL	11	Kb	65	1.0			
LQYIGIFLAI	10	Kb	457	1.0			
LQYIGIFLAIL	11	Kb	56	1.0			
QYIGIFLAI	9	Kd	18	1.0			
QYIGIFLAIL	10	Kd	59	1.0			
QYIGIFLAILL	11	Kd	116	1.0			
IGIFLAIL	8	Kb	142	1.0			
IGIFLAILL	9	Kb	76	1.0			
VGISIIAAI	9	Kb	461	1.1	89256352	hypothetical protein	
ISIIAAIFSYL	11	Kb	76	1.1			L
SIIAAIFSYL	10	Kb	283	1.1			L
IIAAIFSYL	9	Kb	329	1.1			
IAAIFSYL	8	Kb	224	1.1			
IAAIFSYLAAL	11	Kb	63	1.1			
AAIFSYLAAL	10	Kb	42	1.1			
AAIFSYLAALI	11	Kb	728	1.1			
AIFSYLAAL	9	Kb	34	1.1			
AIFSYLAALI	10	Kb	534	1.1			
FSYLAALI	8	Kb	291	1.1			
FSYLAALIAI	10	Db	73	1.1			
FSYLAALIAII	11	Db	478	1.1			
SYLAALIAI	9	Kd	15	1.1			
SYLAALIAII	10	Kd	49	1.1			
SYLAALIAIII	11	Kd	81	1.1			
LAALIAIIIGL	11	Kb	499	1.1			
AALIAIII	8	Db	778	1.1			
AALIAIIIGL	10	Kb	137	1.1			
ISIFLYLFL	9	Kb	45	1.0	89256377	hypothetical membrane protein	L
ISIFLYLFLV	10	Kb	559	1.0			
ISIFLYLFLVI	11	Kb	337	1.0			
SIFLYLFL	8	Kb	54	1.0			L
SIFLYLFLV	9	Kb	843	1.0			
SIFLYLFLVI	10	Kb	536	1.0			
SIFLYLFLVIV	11	Kb	703	1.0			
IFLYLFLVIVM	11	Kb	241	1.0			
FLYLFLVIVM	10	Kb	133	1.0			
FLYLFLVIVMI	11	Kb	650	1.0			
LYLFLVIVM	9	Kb	354	1.0			
LYLFLVIVMI	10	Kd	383	1.0			
LVIVMIYFTSL	11	Kb	140	1.0			
VIVMIYFTSL	10	Kb	49	1.0			
IVMIYFTSL	9	Kb	17	1.0			
VMIYFTSL	8	Kb	33	1.0			
MIYFTSLSNQI	11	Kb	412	1.0			
IYFTSLSNQI	10	Kd	8	1.0			
IYFTSLSNQIL	11	Kd	41	1.0			
YFTSLSNQI	9	Kd	759	1.0			
TSLSNQIL	8	Db	195	1.0			
TSLSNQILL	9	Db	10	1.0			
LSYYGVFVAFI	11	Kb	156	1.0	89256404	amino acid permease	
SYYGVFVAF	9	Kd	758	1.0			
SYYGVFVAFI	10	Kd	79	1.0			
SYYGVFVAFII	11	Kd	137	1.0			
YGVFVAFIIML	11	Kb	671	1.0			
GVFVAFIIML	10	Kb	478	1.0			
VFVAFIIML	9	Kb	280	1.0			
VFVAFIIMLSL	11	Kb	471	1.0			
VAFIIMLSL	9	Kb	46	1.0			
AFIIMLSLTYL	11	Kd	67	1.0			
FIIMLSLTYL	10	Db	212	1.0			
IIMLSLTYL	9	Db	105	1.0			L
IMLSLTYL	8	Db	126	1.0			
LSLTYLNT	8	Kb	159	1.0			
LSLTYLNTYKL	11	Kb	124	1.0			L
LTYLNTYKL	9	Kb	43	1.0			
TYLNTYKL	8	Kd	512	1.0			
IAISFLYF	8	Kb	712	1.0	89256517	hypothetical protein	
IAISFLYFM	9	Db	60	1.0			
IAISFLYFMP	10	Kb	402	1.0			
IAISFLYFMPI	11	Kb	106	1.0			
ISFLYFMP	8	Kb	713	1.0			

Sequence	Length	Predicted allele	Affinity (IC_{50}, nM)	Cluster density	Protein gi#	Protein annotation	Responder[(a)]
ISFLYFMPI	9	Kb	23	1.0			
ISFLYFMPII	10	Kb	55	1.0			L
SFLYFMPII	9	Kb	692	1.0			L
SFLYFMPIISL	11	Kb	59	1.0			L
FLYFMPIISL	10	Kb	60	1.0			L
LYFMPIISL	9	Kb	81	1.0			M
LYFMPIISLFL	11	Kb	701	1.0			
YFMPIISL	8	Kd	770	1.0			
FMPIISLFL	9	Db	216	1.0			
ISLFLGWIFL	10	Kb	168	1.0			
SLFLGWIFL	9	Kb	615	1.0			L
PSVQAYYWL	9	Kb	749	1.0	89256552	amino acid antiporter	L
PSVQAYYWLL	10	Kb	728	1.0			L
SVQAYYWL	8	Kb	82	1.1			
SVQAYYWLL	9	Kb	843	1.1			L
VQAYYWLLTAL	11	Kb	89	1.1			
QAYYWLLTAL	10	Kb	24	1.1			
AYYWLLTAL	9	Kd	19	1.1			
YYWLLTAL	8	Kd	13	1.1			
YWLLTALSTQI	11	Kd	488	1.1			
WLLTALSTQI	10	Kd	878	1.1			
LTALSTQIYSL	11	Kb	515	1.1			
TALSTQIYSL	10	Kb	51	1.1			
TALSTQIYSLM	11	Kb	74	1.1			
LSTQIYSLM	9	Kb	168	1.1			
LSTQIYSLMYL	11	Kb	170	1.1			
STQIYSLM	8	Kb	125	1.1			
STQIYSLMYL	10	Kb	716	1.1			
TQIYSLMYL	9	Kb	111	1.1			
TQIYSLMYLM	10	Kb	297	1.1			
TQIYSLMYLMM	11	Kb	133	1.1			
QIYSLMYL	8	Kb	270	1.1			
QIYSLMYLM	9	Kb	530	1.1			H
QIYSLMYLMM	10	Kb	240	1.1			M
IYSLMYLMM	9	Kb	784	1.1			
YSLMYLMM	8	Db	58	1.1			
YSLMYLMMFFA	11	Db	722	1.1			
LMYLMMFFAA	10	Kb	602	1.1			
LMYLMMFFAAL	11	Kb	11	1.1			L
MYLMMFFAAL	10	Kd	35	1.1			
YLMMFFAAL	9	Kb	120	1.1			
YLMMFFAALKL	11	Kb	978	1.1			
LMMFFAAL	8	Kb	108	1.2			
LMMFFAALKL	10	Kb	206	1.2			
MMFFAALKL	9	Kb	321	1.2			
MMFFAALKLKL	11	Kb	318	1.2			
FIITILYFI	9	Db	119	1.0	89256614	virulence factor MviN	
FIITILYFIAL	11	Db	780	1.0			
IITILYFIAL	10	Kb	158	1.0			
ITILYFIAL	9	Kb	13	1.0			
ITILYFIALFL	11	Kb	149	1.0			
TILYFIAL	8	Kb	191	1.0			L
TILYFIALFL	10	Kb	130	1.0			
TILYFIALFLL	11	Kb	72	1.0			
ILYFIALFL	9	Kb	273	1.0			
ILYFIALFLL	10	Kb	142	1.0			
ILYFIALFLLI	11	Kb	759	1.0			
LYFIALFLL	9	Kb	616	1.0			
IALFLLII	8	Db	848	1.0			
IALFLLIITFL	11	Kb	53	1.0			
ALFLLIITFL	10	Kb	307	1.0			
LFLLIITFL	9	Kd	182	1.0			
LIITFLAI	8	Kb	668	1.0			
IALSAYANI	9	Kb	223	1.0	89256710	major facilitator superfamily (MFS) transport protein	
IALSAYANIM	10	Kb	51	1.0			
LSAYANIMY	9	Dd	670	1.0			
LSAYANIMYYL	11	Kb	403	1.0			L
SAYANIMYYL	10	Kb	21	1.0			
SAYANIMYYLV	11	Db	107	1.0			
AYANIMYYL	9	Kd	693	1.0			
YANIMYYL	8	Db	56	1.0			
YANIMYYLV	9	Db	633	1.0			
YANIMYYLVL	10	Db	73	1.0			
ANIMYYLV	8	Kb	38	1.0			
ANIMYYLVL	9	Kb	150	1.0			
NIMYYLVLSYL	11	Kb	417	1.0			
IMYYLVLSYL	10	Kb	10	1.0			
MYYLVLSYL	9	Kd	27	1.0			
YYLVLSYL	8	Kd	25	1.0			
LSYLSNHFVEL	11	Kb	35	1.0			
SYLSNHFV	8	Kd	46	1.0			
SYLSNHFVEL	10	Kd	43	1.0			
LIFAYPSF	8	Kb	76	1.0			
LIFAYPSFM	9	Kb	269	1.0			
LIFAYPSFML	10	Kb	269	1.0			
LIFAYPSFMLM	11	Kb	208	1.0			
FAYPSFML	8	Db	931	1.0			
FAYPSFMLM	9	Kb	150	1.0			
FAYPSFMLMNM	11	Kb	139	1.0			
AYPSFMLMNM	10	Kb	514	1.0			
FMLMNMGV	8	Db	335	1.0			
FMLMNMGVV	9	Db	6	1.0			
FMLMNMGVVY	10	Db	680	1.0			
FMLMNMGVVYL	11	Db	6	1.0			L
MGVVYLVL	8	Kb	112	1.0			
MGVVYLVLAM	10	Kb	565	1.0			
VVYLVLAM	8	Kb	255	1.0			

Sequence	Length	Predicted allele	Affinity (IC_{50}, nM)	Cluster density	Protein gi#	Protein annotation	Responder[(a)]
VVYLVLAMI	9	Kb	244	1.0			
VVYLVLAMII	10	Kb	500	1.0			
VVYLVLAMIIL	11	Kb	53	1.0			
VYLVLAMI	8	Kd	361	1.0			
YLVLAMIIL	9	Db	618	1.0			
VLAMIILSVPL	11	Kb	613	1.0			
LAMIILSVPL	10	Kb	118	1.0			
LAMIILSVPLM	11	Kb	263	1.0			
AMIILSVPL	9	Kb	97	1.0			
AMIILSVPLM	10	Kb	236	1.0			
MIILSVPL	8	Kb	367	1.0			L
MIILSVPLM	9	Kb	686	1.0			
IILSVPLM	8	Kb	433	1.0			
MEFSVTYFIML	11	Kb	421	1.1	89256727	DoxD-like family protein	
FSVTYFIM	8	Db	26	1.1			
FSVTYFIML	9	Db	47	1.1			
SVTYFIML	8	Kb	782	1.1			
SVTYFIMLFTL	11	Kb	144	1.1			
VTYFIMLFTL	10	Kb	21	1.1			
TYFIMLFTL	9	Kd	61	1.1			
TYFIMLFTLF	10	Kd	977	1.1			
TYFIMLFTLFL	11	Kb	258	1.1			
YFIMLFTL	8	Kd	461	1.1			
FIMLFTLFL	9	Db	643	1.1			
IMLFTLFL	8	Db	133	1.1			
ASLTIAIL	8	Kb	539	1.0	89256785	cysteine/glutathione ABC transporter membrane/ATP-binding component	
TIAILFSFFSL	11	Kb	256	1.0			
IAILFSFF	8	Kb	687	1.0			L
IAILFSFFSL	10	Kb	26	1.0			
AILFSFFSL	9	Kb	73	1.0			
AILFSFFSLSL	11	Kb	96	1.0			L
ILFSFFSL	8	Kb	84	1.0			L
ILFSFFSLSL	10	Kb	39	1.0			L
FSFFSLSL	8	Kb	790	1.0			
FSFFSLSLAL	10	Kb	128	1.0			
FSFFSLSLALL	11	Kb	113	1.0			
SFFSLSLAL	9	Kb	667	1.0			
SFFSLSLALL	10	Kb	619	1.0			L
FSLSLALL	8	Kb	138	1.0			
FSLSLALLTFI	11	Db	56	1.0			
LSLALLTFI	9	Db	393	1.0			
LSLALLTFIAL	11	Kb	41	1.0			
SLALLTFIAL	10	Kb	658	1.0			L
LALLTFIAL	9	Kb	21	1.0			
LALLTFIALL	10	Kb	42	1.0			
LALLTFIALLL	11	Kb	136	1.0			
ALLTFIAL	8	Kb	419	1.0			
ALLTFIALL	9	Kb	304	1.0			
ALLTFIALLL	10	Kb	987	1.0			
IALLLIGFVI	10	Db	643	1.0			
IALLLIGFVIP	11	Kb	920	1.0			
ALLLIGFVIPL	11	Kb	432	1.0			
LLLIGFVIPL	10	Kb	215	1.2			
LLIGFVIPL	9	Kb	297	1.2			
LSSYILQYIL	10	Kb	812	1.1	89256788	sulfate permease family protein	
LSSYILQYILL	11	Kb	196	1.1			
SSYILQYI	8	Kb	217	1.1			
SSYILQYIL	9	Kb	41	1.1			
SSYILQYILL	10	Kb	16	1.1			
SYILQYIL	8	Kd	200	1.1			
SYILQYILL	9	Kb	131	1.1			
YILQYILL	8	Kb	97	1.1			
LQYILLCVI	9	Kb	388	1.1			
LQYILLCVIM	10	Kb	91	1.1			
QYILLCVI	8	Kd	12	1.1			L
QYILLCVIM	9	Kd	186	1.1			
KSNSFFYIFI	10	Db	888	1.0	89256811	cell division protein	
NSFFYIFI	8	Kb	551	1.0			
NSFFYIFISV	10	Kb	287	1.0			
NSFFYIFISVV	11	Kb	913	1.0			
SFFYIFISV	9	Kb	365	1.0			
SFFYIFISVVL	11	Kb	167	1.0			L
FFYIFISV	8	Kb	196	1.0			
FFYIFISVVL	10	Kb	287	1.0			
FFYIFISVVLL	11	Kb	242	1.0			
FYIFISVV	8	Kd	56	1.0			L
FYIFISVVL	9	Kd	81	1.0			
FYIFISVVLL	10	Kd	175	1.0			
FYIFISVVLLL	11	Kd	329	1.0			
YIFISVVLL	9	Kb	818	1.0			L
IFISVVLL	8	Kb	479	1.0			L
ISVVLLLIAI	10	Db	345	1.0			
ISVVLLLIAIL	11	Kb	334	1.0			
SVVLLLIAI	9	Db	870	1.0			
SVVLLLIAIL	10	Kb	266	1.0			
VVLLLIAIL	9	Kb	84	1.0			
IAILQYNL	8	Kb	245	1.0			
MIFIIPIL	8	Kb	364	1.1	89256851	hypothetical protein	
IIPILFSYSM	10	Kb	61	1.1			
IIPILFSYSMI	11	Kb	610	1.1			
IPILFSYSM	9	Kb	175	1.1			M
ILFSYSMI	8	Kb	133	1.1			
ILFSYSMIIVI	11	Kb	706	1.1			
FSYSMIIV	8	Kb	740	1.1			
FSYSMIIVI	9	Db	63	1.1			
FSYSMIIVITI	11	Db	100	1.1			
SYSMIIVI	8	Kd	536	1.1			

Sequence	Length	Predicted allele	Affinity (IC_{50}, nM)	Cluster density	Protein gi#	Protein annotation	Responder[a]
SYSMIIVITI	10	Kd	542	1.1			
SYSMIIVITIL	11	Kd	111	1.1			
YSMIIVITI	9	Db	109	1.1			
YSMIIVITIL	10	Db	575	1.1			
SMIIVITI	8	Db	510	1.1			
SMIIVITIL	9	Kb	435	1.1			
MIIVITIL	8	Kb	874	1.1			
IVLVYIVYI	9	Kb	381	1.0	89256861	PerM family protein	
IVLVYIVYIL	10	Kb	196	1.0			
LVYIVYIL	8	Kb	199	1.0			
LVYIVYILFL	10	Kb	79	1.0			
LVYIVYILFLI	11	Kb	379	1.0			
VYIVYILF	8	Kb	449	1.0			
YIVYILFLIAL	11	Kb	413	1.0			
IVYILFLIAL	10	Kb	11	1.0			
IVYILFLIALL	11	Kb	20	1.0			
VYILFLIAL	9	Kb	38	1.0			
VYILFLIALL	10	Kb	94	1.0			
YILFLIALL	9	Kb	289	1.0			
YILFLIALLSL	11	Kb	303	1.0			
ILFLIALL	8	Kb	356	1.0			
ILFLIALLSL	10	Kb	194	1.0			
IALLSLIFV	9	Db	19	1.0			
IALLSLIFVL	10	Db	49	1.0			
IALLSLIFVLL	11	Kb	50	1.0			
ALLSLIFVL	9	Kb	962	1.0			
ALLSLIFVLL	10	Kb	484	1.0			
LSLIFVLL	8	Kb	32	1.0			
LSLIFVLLPI	10	Kb	416	1.0			
LSLIFVLLPII	11	Kb	498	1.0			
SSYISVRI	8	Kb	479	1.0	89256892	Phospho-N-acetylmuramoyl-pentapeptide transferase	
SSYISVRII	9	Kb	679	1.0			
SSYISVRIIM	10	Kb	64	1.0			L
SSYISVRIIMI	11	Kb	175	1.0			
SYISVRII	8	Kd	27	1.0			
SYISVRIIM	9	Kd	39	1.0			H
SYISVRIIMI	10	Kd	22	1.0			
ISVRIIMISI	10	Kb	655	1.0			
VRIIMISITSL	11	Kb	201	1.0			
RIIMISITSL	10	Kb	800	1.0			L
IIMISITSL	9	Kb	115	1.0			L
IIMISITSLL	10	Kb	357	1.0			
IMISITSL	8	Kb	472	1.0			
IMISITSLL	9	Kb	181	1.0			
ISITSLLI	8	Db	366	1.0			
ISITSLLITL	10	Db	55	1.0			M
SITSLLITL	9	Kb	563	1.0			M
ITSLLITLAL	10	Kb	602	1.0			
TSLLITLAL	9	Kb	86	1.0			
SSTTGYITI	9	Db	168	1.0	89256896	hypothetical protein	
SSTTGYITIIL	11	Kb	886	1.0			L
TTGYITIIL	9	Kb	748	1.0			L
TGYITIIL	8	Kb	365	1.0			
TGYITIILNL	10	Kb	52	1.0			
TGYITIILNLL	11	Kb	24	1.0			
GYITIILNL	9	Kd	112	1.0			
GYITIILNLL	10	Kd	31	1.0			
ITIILNLL	8	Kb	474	1.0			
TIILNLLYA	9	Db	371	1.0			
TIILNLLYAQL	11	Kb	114	1.0			
IILNLLYAQL	10	Kb	58	1.0			
ILNLLYAQL	9	Kb	642	1.0			M
LNLLYAQL	8	Kb	5	1.0			M
LNLLYAQLFNL	11	Kb	309	1.0			M
LLYAQLFNL	9	Kb	270	1.0			
LYAQLFNL	8	Kd	360	1.0			
LYAQLFNLSSL	11	Kd	10	1.0			M
YAQLFNLSSL	10	Kb	408	1.0			
AQLFNLSSL	9	Kb	35	1.0			
FNLSSLGYI	9	Db	248	1.0			
FNLSSLGYISI	11	Db	447	1.0			
LSSLGYISI	9	Kb	768	1.0			
LSSLGYISIPL	11	Kb	206	1.0			
SSLGYISI	8	Kb	69	1.0			
SSLGYISIPL	10	Kb	52	1.0			
LGYISIPL	8	Kb	201	1.0			
LGYISIPLAFI	11	Kb	381	1.0			
GYISIPLAFI	10	Kd	32	1.0			
GYISIPLAFII	11	Kd	55	1.0			
ISIPLAFI	8	Kb	950	1.0			
ISIPLAFIIL	10	Kb	201	1.0			
ISIPLAFIILL	11	Kb	118	1.0			
SIPLAFIIL	9	Kb	296	1.0			
SIPLAFIILL	10	Kb	159	1.0			
IPLAFIIL	8	Kb	806	1.0			
IPLAFIILLVL	11	Kb	458	1.0			
KNPSRNVP	8	Dd	932	1.0	89256917	amino acid transporter protein, fragment	
KNPSRNVPL	9	Dd	37	1.0			
KNPSRNVPLA	10	Dd	596	1.0			
KNPSRNVPLAI	11	Dd	298	1.0			
NVPLAIILSL	10	Kb	771	1.0			
VPLAIILSL	9	Kb	441	1.0			
VPLAIILSLAL	11	Kb	855	1.0			
LAIILSLAL	9	Kb	324	1.0			
LAIILSLALVL	11	Kb	657	1.0			
AIILSLALVLL	11	Kb	617	1.0			
IILSLALV	8	Kb	414	1.0			

Sequence	Length	Predicted allele	Affinity (IC_{50}, nM)	Cluster density	Protein gi#	Protein annotation	Responder[(a)]
IILSLALVL	9	Kb	562	1.0			
IILSLALVLL	10	Kb	249	1.0			L
IILSLALVLLL	11	Kb	457	1.0			
LSLALVLL	8	Kb	484	1.0			
LSLALVLLL	9	Kb	290	1.0			
LSLALVLLLYM	11	Kb	216	1.0			
LALVLLLYM	9	Db	58	1.0			
LALVLLLYMGL	11	Kb	141	1.0			
ALVLLLYMGL	10	Kb	726	1.0			
LVLLLYMGL	9	Kb	50	1.0			
VLLLYMGL	8	Kb	77	1.0			
LLYMGLQYAFM	11	Kb	129	1.0			
LYMGLQYAFM	10	Kd	631	1.0			
YMGLQYAFM	9	Db	25	1.0			
MGLQYAFM	8	Kb	27	1.0			
MGLQYAFMQAV	11	Kb	513	1.0			
LQYAFMQA	8	Kb	655	1.0			
LQYAFMQAV	9	Kb	156	1.0			
LQYAFMQAVP	10	Kb	338	1.0			
QYAFMQAV	8	Kd	120	1.0			
LMLTLSIVSL	10	Kb	111	1.0	89256946	major facilitator superfamily (MFS) transport protein	
LTLSIVSLAM	10	Kb	538	1.0			
LTLSIVSLAML	11	Kb	279	1.0			
LSIVSLAM	8	Db	902	1.0			
LSIVSLAML	9	Kb	436	1.0			
LSIVSLAMLL	10	Kb	603	1.0			
SIVSLAML	8	Kb	171	1.0			
VSLAMLLAI	9	Kb	378	1.0			
VSLAMLLAII	10	Kb	478	1.0			
VSLAMLLAIIL	11	Kb	81	1.0			
LAMLLAIIL	9	Kb	247	1.0			
AMLLAIIL	8	Db	304	1.0			
AMLLAIILYV	10	Db	846	1.0			
MLLAIILYVPM	11	Kb	282	1.0			
LLAIILYVPM	10	Kb	795	1.0			
LAIILYVPM	9	Kb	21	1.0			
LAIILYVPMSL	11	Kb	41	1.0			
AIILYVPM	8	Kb	31	1.0			M
AIILYVPMSL	10	Kb	101	1.0			
IILYVPMSL	9	Kb	51	1.0			
IILYVPMSLSM	11	Kb	108	1.0			H
ILYVPMSL	8	Kb	129	1.0			
ILYVPMSLSM	10	Kb	295	1.0			
LYVPMSLSM	9	Kd	79	1.1			
LYVPMSLSMF	10	Kd	338	1.0			
MSLSMFTVL	9	Kb	68	1.1			
LSMFTVLYFL	10	Db	251	1.1			
LSMFTVLYFLL	11	Kb	123	1.1			
SMFTVLYFLL	10	Kb	191	1.0			L
IIGIFLMFL	9	Kb	723	1.0	89256977	potassium uptake protein	
IIGIFLMFLSL	11	Kb	217	1.0			
IGIFLMFL	8	Kb	581	1.0			
IGIFLMFLSL	10	Kb	34	1.0			
GIFLMFLSL	9	Kb	126	1.0			
GIFLMFLSLTM	11	Kb	392	1.0			
IFLMFLSL	8	Kb	398	1.0			L
IFLMFLSLTM	10	Kb	243	1.0			
IFLMFLSLTML	11	Kb	222	1.0			
LMFLSLTM	8	Db	748	1.0			H
LMFLSLTML	9	Kb	115	1.0			
LMFLSLTMLSP	11	Kb	700	1.0			H
MFLSLTML	8	Kd	493	1.0			
MFLSLTMLSPL	11	Kd	201	1.0			
LSLTMLSPL	9	Kb	58	1.0			L
LSLTMLSPLL	10	Kb	130	1.0			
TMLSPLLVDYI	11	Db	178	1.0			
LSPLLVDYI	9	Db	177	1.0			
LPYWLAVAI	9	Kb	295	1.0	89257033	cytochrome oxidase bd-II, subunit II	
LAVAIFILTYI	11	Db	905	1.0			
VAIFILTYI	9	Db	240	1.0			
VAIFILTYISM	11	Kb	21	1.0			M
AIFILTYISM	10	Kb	43	1.0			
AIFILTYISML	11	Kb	149	1.0			
IFILTYISM	9	Kb	65	1.0			M
IFILTYISML	10	Kb	230	1.0			
FILTYISM	8	Kb	44	1.0			
FILTYISML	9	Kb	529	1.0			
FILTYISMLTL	11	Db	753	1.0			
LTYISMLTL	9	Kb	88	1.0			
LTYISMLTLI	10	Kb	532	1.0			
TYISMLTL	8	Kd	9	1.0			
TYISMLTLI	9	Kd	1	1.0			
TYISMLTLIF	10	Kd	609	1.0			
ISMLTLIFPYI	11	Db	70	1.0			
SMLTLIFPYI	10	Db	89	1.0			
SMLTLIFPYII	11	Db	118	1.0			
LTLIFPYI	8	Kb	204	1.0			
LIFPYIIPYQI	11	Kb	708	1.1			
AALALAIIQL	10	Kb	126	1.0	89257093	hypothetical protein	M
AALALAIIQLL	11	Kb	176	1.1			
LALAIIQLL	9	Kb	157	1.1			
LAIIQLLIYL	10	Db	101	1.1			
AIIQLLIYL	9	Kb	82	1.1			
IIQLLIYL	8	Kb	686	1.1			
IQLLIYLTNSA	11	Kb	837	1.1			
LLIYLTNSAL	10	Kb	247	1.1			
LLIYLTNSALL	11	Kb	329	1.1			

Sequence	Length	Predicted allele	Affinity (IC_{50}, nM)	Cluster density	Protein gi#	Protein annotation	Responder[(a)]
LIYLTNSAL	9	Kb	79	1.1			
LIYLTNSALL	10	Kb	124	1.1			
IYLTNSAL	8	Kd	12	1.3			
IYLTNSALL	9	Kd	327	1.3			
IYLTNSALLFI	11	Db	113	1.1			
TNSALLFITL	10	Kb	868	1.3			
NSALLFITL	9	Kb	268	1.3			
SALLFITL	8	Kb	83	1.3			
SALLFITLRFI	11	Db	122	1.3			
LFITLRFI	8	Kd	443	1.0			
CYHKNTSNNYL	11	Kd	10	1.0			
NNYLIYVYFSV	11	Kb	586	1.0			
LIYVYFSV	8	Kb	14	1.0			M
LIYVYFSVI	9	Kb	325	1.0			
LIYVYFSVIFL	11	Kb	103	1.0			
IYVYFSVI	8	Kd	198	1.0			
IYVYFSVIFL	10	Kb	291	1.0			
YVYFSVIFL	9	Db	107	1.0			M
YVYFSVIFLAI	11	Kb	784	1.0			
VYFSVIFL	8	Kb	265	1.0			
VYFSVIFLAI	10	Kd	290	1.0			
VYFSVIFLAIL	11	Kb	214	1.0			
FSVIFLAI	8	Db	362	1.0			
FSVIFLAIL	9	Kb	194	1.0			
FSVIFLAILL	10	Kb	245	1.0			
SVIFLAIL	8	Kb	499	1.0			
SVIFLAILL	9	Kb	166	1.0			
VIFLAILL	8	Kb	301	1.0			
AILLAQTAKFL	11	Kb	945	1.0			
ILLAQTAKFL	10	Kb	829	1.0			L
SSMQTVFYV	9	Db	60	1.0			
TVFYVTAII	9	Kb	595	1.0			
TVFYVTAIIYM	11	Kb	64	1.0			H
VFYVTAII	8	Kb	503	1.0			
VFYVTAIIYM	10	Kb	265	1.0			H
FYVTAIIYM	9	Db	86	1.0			
FYVTAIIYMFL	11	Kd	23	1.0			
VTAIIYMFL	9	Kb	427	1.0			
TAIIYMFL	8	Kb	187	1.0			
TAIIYMFLVVI	11	Kb	563	1.0			
AIIYMFLVVI	10	Kb	785	1.0			
IIYMFLVV	8	Kb	52	1.0			
IIYMFLVVI	9	Kb	61	1.0			
IIYMFLVVIYI	11	Kb	67	1.0			
IYMFLVVI	8	Kd	498	1.1			
IYMFLVVIYI	10	Kd	673	1.0			
YMFLVVIYI	9	Db	95	1.1			
YMFLVVIYICL	11	Db	200	1.1			
FLVVIYICL	9	Db	609	1.1			
LVVIYICL	8	Kb	395	1.1			
LVVIYICLL	9	Kb	206	1.1			
LVVIYICLLYL	11	Kb	394	1.1			
VVIYICLLYL	10	Kb	302	1.1			
VIYICLLYL	9	Kb	78	1.1			
IYICLLYL	8	Kd	299	1.0			L
IYICLLYLFDL	11	Kd	638	1.0			L
ICLLYLFDL	9	Kb	312	1.1			
FSVVLRQHVYI	11	Db	46	1.0	89257107	toxin secretion ABC transporter ATP-binding protein	
SVVLRQHVYI	10	Db	243	1.0			
VVLRQHVYI	9	Kb	541	1.0			
VVLRQHVYIGL	11	Kb	86	1.0			M
RQHVYIGL	8	Kb	203	1.0			
RQHVYIGLTYI	11	Db	869	1.0			
QHVYIGLTYI	10	Kd	885	1.0			
HVYIGLTYIFI	11	Db	732	1.0			
VYIGLTYI	8	Kd	19	1.0			
VYIGLTYIFI	10	Kd	400	1.0			
YIGLTYIFI	9	Db	108	1.0			
IGLTYIFI	8	Kb	137	1.0			
IGLTYIFINI	10	Kb	471	1.0			
IGLTYIFINIL	11	Kb	56	1.0			
LTYIFINI	8	Kb	10	1.0			
LTYIFINIL	9	Kb	12	1.0			
LTYIFINILL	10	Kb	28	1.0			
LTYIFINILLL	11	Kb	25	1.0			
TYIFINIL	8	Kd	70	1.0			
TYIFINILL	9	Kd	282	1.0			
TYIFINILLL	10	Kd	894	1.0			
YIFINILLL	9	Kb	667	1.0			
SQVSRRYLL	9	Kb	448	1.2	89257127	aromatic amino acid transporter of the HAAAP family	
VSRRYLLF	8	Kb	468	1.2			
RRYLLFFSI	9	Kb	535	1.2			
RRYLLFFSII	10	Kb	864	1.2			
RYLLFFSI	8	Kd	41	1.2			
RYLLFFSII	9	Kd	42	1.2			
RYLLFFSIIYL	11	Kd	162	1.2			
YLLFFSIIYL	10	Kb	389	1.2			
YLLFFSIIYLL	11	Kb	914	1.2			
LLFFSIIYL	9	Kb	128	1.2			
LLFFSIIYLL	10	Kb	293	1.2			
LLFFSIIYLLL	11	Kb	250	1.2			
LFFSIIYLL	9	Kb	798	1.2			
LFFSIIYLLL	10	Kb	654	1.2			
LFFSIIYLLLL	11	Kb	737	1.2			
FSIIYLLL	8	Kb	17	1.2			
FSIIYLLLL	9	Db	182	1.2			
SIIYLLLLYSL	11	Kb	78	1.2			

Sequence	Length	Predicted allele	Affinity (IC_{50}, nM)	Cluster density	Protein gi#	Protein annotation	Responder[(a)]
IIYLLLLYSL	10	Kb	14	1.2			
IIYLLLLYSLI	11	Kb	140	1.2			
IYLLLLYSL	9	Kb	70	1.2			
IYLLLLYSLI	10	Kd	74	1.2			
FSLYLNYSSI	10	Kb	220	1.0	89257149	hypothetical protein	
SLYLNYSSI	9	Kb	48	1.0			
SLYLNYSSII	10	Kb	378	1.0			
SLYLNYSSIIP	11	Kb	763	1.0			
LYLNYSSI	8	Kd	31	1.0			
LYLNYSSII	9	Kd	320	1.0			
SSIIPTYFL	9	Db	57	1.0			
SSIIPTYFLL	10	Kb	245	1.0			
SSIIPTYFLLL	11	Kb	298	1.0			
SIIPTYFL	8	Kb	688	1.0			
SIIPTYFLL	9	Kb	420	1.0			
SIIPTYFLLL	10	Kb	510	1.0			
IIPTYFLL	8	Kb	42	1.0			
IIPTYFLLL	9	Kb	237	1.0			
TYFLLLFSSEI	11	Kd	38	1.0			
YFLLLFSSEI	10	Kd	228	1.0			
SFLTNYAGFI	10	Db	384	1.0	89257164	Competence-related protein	
ISYWSYFSF	9	Kb	145	1.0			M
ISYWSYFSFF	10	Kb	462	1.0			H
SYWSYFSFF	9	Kd	807	1.0			L
SYWSYFSFFSL	11	Kd	16	1.0			H
WSYFSFFSL	9	Kb	24	1.0			M
WSYFSFFSLV	10	Kb	416	1.0			
WSYFSFFSLVI	11	Db	555	1.0			
SYFSFFSL	8	Kd	13	1.0			
SYFSFFSLV	9	Kd	42	1.0			
SYFSFFSLVI	10	Kd	145	1.0			
SYFSFFSLVII	11	Kd	27	1.0			
FSFFSLVI	8	Db	309	1.0			
FSFFSLVII	9	Db	197	1.4			
FSFFSLVIII	10	Db	256	1.0			
FSFFSLVIIIL	11	Kb	137	1.0			
SFFSLVIIIL	10	Kb	555	1.4			
FSLVIIIL	8	Db	178	1.4			
FSLVIIILGII	11	Db	397	1.4	89257164	Competence-related protein	
RIFVFSYT	8	Kb	228	1.0			
RIFVFSYTFM	10	Kb	704	1.0			
RIFVFSYTFML	11	Kb	559	1.0			
IFVFSYTFM	9	Db	621	1.0			
IFVFSYTFML	10	Kb	630	1.0			
FVFSYTFM	8	Kb	65	1.0			
FVFSYTFML	9	Kb	172	1.0			
FSYTFMLY	8	Kb	68	1.0			
FSYTFMLYAFI	11	Db	35	1.0			
SYTFMLYAFI	10	Kd	87	1.0			
YTFMLYAFI	9	Kb	471	1.0			
FMLYAFIAGTI	11	Db	237	1.0			
MLYAFIAGTI	10	Kb	642	1.0			
LYAFIAGTI	9	Kd	45	1.0			
LYAFIAGTII	10	Kd	63	1.0			
YAFIAGTIIAI	11	Db	971	1.0			
AFIAGTII	8	Kd	795	1.0			
ATASFPQKYL	10	Kb	469	1.0	89257191	hypothetical protein	
ASFPQKYLQI	10	Kb	606	1.0			
ASFPQKYLQII	11	Kb	937	1.0			
QKYLQIIYI	9	Kb	745	1.0			
QKYLQIIYIL	10	Kb	316	1.0			L
QKYLQIIYILM	11	Kb	136	1.0			
KYLQIIYI	8	Kd	30	1.0			
KYLQIIYIL	9	Kd	155	1.0			
KYLQIIYILM	10	Kb	788	1.0			M
LQIIYILM	8	Kb	419	1.0			M
LQIIYILMM	9	Kb	314	1.0			
IIYILMMFI	9	Kb	564	1.0			
IIYILMMFII	10	Kb	532	1.0			
IYILMMFI	8	Kd	34	1.0			
IYILMMFII	9	Kd	72	1.0			
IYILMMFIIAI	11	Kd	57	1.0			
YILMMFII	8	Db	850	1.0			
YILMMFIIAI	10	Kb	718	1.0			
ILMMFIIAI	9	Kb	279	1.0			
ILMMFIIAITM	11	Kb	113	1.0			
LMMFIIAITM	10	Kb	111	1.0			
MMFIIAITM	9	Kb	92	1.0			
MMFIIAITMI	10	Kb	795	1.0			
MFIIAITMI	9	Kd	747	1.0			
