# Supplementary material for: Whole-Genome Immunoinformatic Analysis of F. tularensis: Predicted CTL Epitopes Clustered in Hotspots Are Prone to Elicit a T-Cell Response
Source: PLoS One. 2011 May 20;6(5):e20050. doi: 10.1371/journal.pone.0020050 (PMC3098878; doi:10.1371/journal.pone.0020050)
Supplement: Table S2 — List of 500 peptides selected by the random-based approach. The affinity provided is the value predicted for a particular responder sequence by the NetMHC3.0 program. Cluster density is the number of predicted CTL epitopes in the responder parental cluster, divided by the parental cluster length. The gi number and annotation of the source protein are according to the F. tularensis holarctica LVS sequence deposited at the NCBI (GenBank accession AM233362); (a) Magnitude of T-cell response for each identified epitope is indicated as follows (expressed in SFC/million cells): L (low) - 7–19; M (medium) - 20–34; H (high) - 35 and above. (PDF) [file pone.0020050.s002.pdf]

Supplement Table 2: Compilation of data for 500 random-based selected putative MHC binders (Subset II)

Sequence	Length	Predicted allele	Affinity (IC_{50}, nM)	Cluster density	Protein gi#	Protein annotation	Responder
SIYLDNPNM	9	Kb	192	0.2	89255451	DNA polymerase III, beta chain	
RALYTQVLKFL	11	Kb	475	0.4	89255455	indolepyruvate decarboxylase	
QYFSKSVL	8	Kd	30	0.5	89255464	propionate kinase	
IKYLNTLMNL	10	Kb	49	0.3	89255465	phosphate acetyltransferase	
VVLNRFDKL	9	Kb	65	0.6	89255469	Proline/betaine transporter, MFS transport protein	
LIPLSISLL	9	Kb	499	0.5			
FLLSFFTIL	9	Kb	374	0.5	89255471	serine transporter	
INIAKQHLLPL	11	Kb	200	0.3	89255472	hypothetical protein	
QTPLKLVSAL	10	Kb	291	0.5	89255476	Carbamoyl-phosphate synthase large chain	
VHFDNTIPL	9	Kb	61	0.3	89255480	Dihydroorotase	
TIFMYFALL	9	Kb	31	NO	89255484	hypothetical membrane protein	
IAIIINDAL	9	Kb	254	0.5			
IALELKDIVI	10	Db	434	NO	89255485	HlyD family secretion protein	
GTGSGNHVT	9	Dd	242	0.3	89255491	Transglutaminase-like superfamily domain protein	
AFITGGFGAI	10	Kd	121	0.2	89255498	hypothetical protein	
SSFDLLPTL	9	Kb	15	0.4	89255505	aromatic amino acid transporter of the HAAAP family	
LFFLVNCSI	9	Kd	157	0.5	89255508	hypothetical membrane protein	
VAYTVYFPGL	10	Kb	12	0.2	89255516	hypothetical membrane protein	
IDPEYLYTHM	10	Kb	467	0.4			
AYIGMIKL	8	Kd	170	0.5	89255522	Transposase	
INFASNIVL	9	Kb	441	NO	89255526	conserved hypothetical protein	
FSIWSVFM	8	Db	64	0.2	89255530	hypothetical membrane protein	
LIFWFWTFSI	10	Kb	183	0.2			
STLKQISKL	9	Kb	177	0.3	89255536	tryptophan synthase alpha chain	
GRGDKDIFT	9	Dd	489	NO	89255537	tryptophan synthase beta chain	
ITPFNFSDGIL	11	Kb	249	0.4	89255538	Carboxypeptidase,fragment	
LIFFISAIL	9	Kb	332	0.5	89255545	major facilitator superfamily (MFS) transport protein, fragment	
LSNLELYPI	9	Db	452	0.2	89255550	intracellular growth locus, subunit C	
HSYGFKHSI	9	Kb	386	0.3	89255551	intracellular growth locus, subunit D	
VMLKVAQL	8	Kb	45	0.2	89255553	conserved hypothetical protein	
LSYINAFL	8	Kb	79	0.1			
YYIPTEENF	9	Kd	295	0.4			
ANFFARPVFKL	11	Kb	324	0.3			
CYQTISLI	8	Kd	28	0.5	89255556	conserved hypothetical protein	
VSLINYVINSV	11	Kb	86	0.4	89255562	conserved hypothetical protein	
SAATNEAI	8	Db	425	NO			
SMITSFLIFF	10	Db	500	0.4			
EYIQSNNDDI	10	Kd	44	NO	89255563	conserved hypothetical protein	
ENFFFDNLVSL	11	Kb	106	0.2			
KYLSTKINF	9	Kd	226	0.5	89255570	ferrous iron transport protein	
CGKTTIFNAL	10	Kb	414	0.3			
TSINKGLPIL	10	Kb	226	NO	89255576	Carboxylesterase/phospholipase family protein	
NIYMTTVDL	9	Kb	392	0.4	89255584	hypothetical protein	
VAYFQIMILP	10	Kb	117	0.6	89255596	major facilitator superfamily (MFS) transport protein, fragment	
QYNILKFL	8	Kd	155	0.2	89255601	DNA/RNA helicase	
YNVQNATACI	10	Db	152	0.3	89255606	UDP-N-acetylmuramate--alanine ligase	
VIITNIDLDHM	11	Db	191	0.1			
FVPYQSSPL	9	Dd	107	0.4	89255617	Oligoribonuclease	
IVVFLLILL	9	Kb	122	0.3	89255620	major facilitator superfamily (MFS) transport protein, fragment	
LGLFLFHFI	9	Kb	423	0.7			
LIYSFRMIGL	10	Kb	24	0.4	89255624	major facilitator superfamily (MFS) transport protein	
ADYQWLVYL	9	Kb	64	0.6			
YMMPNYKPGDM	11	Db	112	0.3	89255625	Cytochrome O ubiquinol oxidase subunit II	
YAMTGMTTQL	10	Db	114	0.3			
QIPHSYAIAM	10	Dd	445	0.4	89255629	Protoheme IX farnesyltransferase	
TGNHNQIII	9	Db	498	NO	89255632	Pyridoxal/pyridoxine/pyridoxamine kinase	
SVYNHFQELL	10	Kb	20	0.5	89255634	methanol dehydrogenase regulatory protein	
TNLDQKVSGL	10	Kb	324	0.2	89255640	conserved hypothetical membrane protein	
VAEIFPHL	8	Kb	335	NO			
CNHVMYHLL	9	Kb	322	0.3	89255641	Pyrrolidone-carboxylate peptidase	
KAHLNDAKDTI	11	Db	35	0.3	89255644	Valyl-tRNA synthetase	
FNPYGYGAV	9	Dd	138	0.3	89255655	amino acid permease	
YTSFNTWFI	9	Db	31	0.4	89255665	phosphatidylglycerophosphate synthetase	
VRAERYVKHPL	11	Kb	430	NO	89255679	30S ribosomal protein S17	
AVLLNANGQPI	11	Db	71	0.2	89255680	50S ribosomal protein L14	
SAVYPKFIEL	10	Kb	271	0.7	89255690	preprotein translocase, subunit Y, membrane protein	
DHPDASTL	8	Dd	424	0.2	89255697	Transposase	
ILYLPPYSPDL	11	Kb	138	0.4	89255699	Transposase	
KYEEGMTEF	9	Kd	298	0.2			
AAVTFLLPTSL	11	Kb	126	0.7	89255706	Glutamate/gamma-aminobutyrate anti-porter	
VVFFAVKTAL	10	Kb	78	0.4	89255708	conserved hypothetical membrane protein	**H**
NAPAGDLYI	9	Dd	86	NO	89255713	heat shock protein, hsp40	
TSFGFHIV	8	Kb	239	0.3	89255715	aromatic amino acid transporter of the HAAAP family	
EYNSVKSNNNI	11	Kd	13	0.2	89255716	hypothetical protein	
RNPTYVKV	8	Kb	56	0.4	89255720	hypothetical membrane protein	
IGLLTTKSI	9	Kb	247	0.8	89255729	hypothetical membrane protein	
FSFVIMVML	9	Kb	328	0.7			
FGYKYREDNAI	11	Kb	232	0.3	89255732	hypothetical protein	
KSVQNPDL	8	Db	239	0.2	89255733	Transposase	
IGFAADTPAGL	11	Kb	98	0.1	89255741	pyruvate dehydrogenase, E2 component	
IIFCVIMFL	9	Kb	280	0.6	89255749	Hypothetical protein yieG	
SYLNNVFGGL	10	Kd	47	0.7	89255750	L-aspartate-beta-decarboxylase	
HIYLRGAKAL	10	Kb	389	0.3	89255754	chorismate mutase	
ASLLFAFLAII	11	Kb	367	0.6	89255755	CDP-alcohol phosphatidyltransferase	
IVYSSYSGGSM	11	Kb	38	0.5	89255760	TolB protein precursor	
DGYNKHVVL	9	Kb	115	0.2			
SNLYARYGRL	10	Kb	143	0.2			
KIYLIGFFAL	10	Kb	275	0.7	89255761	hypothetical lipoprotein	
SQLNRWRKL	9	Kb	227	0.3	89255775	hypothetical protein	
SYLVIFTI	8	Kd	16	0.4	89255776	4-hydroxybenzoate octaprenyltransferase	

Sequence	Length	Predicted allele	Affinity (IC_{50}, nM)	Cluster density	Protein gi#	Protein annotation	Responder
LSLLLLIIL	9	Kb	79	1			
VTFSMYDKI	9	Kb	188	0.4	89255781	heat shock protein HtpX	
LNLFTMVLSNM	11	Kb	373	0.7	89255781		
KYMDYEQTTL	10	Kd	35	0.6	89255782	LemA-like protein	
VYVVMTYNKL	10	Kd	253	0.2			
TAFYKLCGMSM	11	Kb	31	0.4	89255794	superoxide dismuate (Cu-Zn) precusor	
TGAFYVIAKL	10	Kb	154	0.4	89255798	aspartate transaminase	
IVYFAQSLTV	10	Kb	457	0.7	89255799	cation transporter	
AMDSLQSSL	9	Kb	493	0.4	89255801	Type IV pili fiber building block protein	
VAISGILGQIL	11	Kb	492	0.2	89255804	conserved hypothetical membrane protein	
VVELSQKYITL	11	Kb	429	0.4	89255807	Fusion protein PurC/PurD	
FSRLFKSV	8	Kb	324	0.2	89255811	Transposase	
SGFDYFYLL	9	Kb	37	0.2	89255820	Purine/pyrimidine phosphoribosyl transferase family protein	
MSFLVAIV	8	Kb	102	NO	89255825	GTP-binding protein	
LAQSNLENAL	10	Kb	273	0.2	89255830	Oligopeptidase A	
FMGRKPNEDAL	11	Db	408	0.3			
AEYIIILPLL	10	Kb	150	0.5	89255835	Type IV pili glycosylation protein	
MPFMHYTVLNL	11	Kb	424	0.3			
YSLLNDCV	8	Db	55	0.5	89255840	hypothetical protein	
IAAIVLIYL	9	Kb	222	0.8	89255845	lipoprotein signal peptidase II	
DALLNAPGYL	10	Kb	483	0.2	89255848	NAD-dependent malic enzyme	
ANFVGAGL	8	Kb	323	0.2	89255853	major facilitator superfamily (MFS) transport protein	
IAYQFLIEGEI	11	Kb	96	0.4	89255857	conserved hypothetical membrane protein	
TNILLKVLIPL	11	Kb	156	0.3			
SKYKIKTADLM	11	Kb	207	0.2	89255871	conserved hypothetical protein	
AAMRYTESRL	10	Kb	131	0.4	89255872	DNA topoisomerase IV subunit A	
IGYSLTTQQV	10	Kb	192	0.2	89255876	soluble lytic murein transglycosylase	
AYHSNEIRQHI	11	Kd	62	NO	89255878	Transposase	
FVFINANTI	9	Db	115	0.7	89255879	hypothetical protein	
SKLCYQGL	8	Kb	256	0.3	89255882	DNA polymerase III alpha subunit	
FGTNNYIIEI	10	Db	498	0.2			
TGMDASNASM	10	Kb	286	0.4	89255889	glycine cleavage system P protein, subunit 1	
IDTNFYPL	8	Kb	91	0.3	89255890	glycine cleavage system P protein, subunit 2	
FTHLNATHQV	10	Db	361	0.2	89255892	pullulonase	
QYKSHSLL	8	Kd	484	0.6	89255895	Glucose-1-phosphate adenylyltransferase	
YGVLNGVDPQI	11	Db	481	0.2	89255896	glycogen synthase	
NGPLYYRDVFL	11	Kb	467	0.3	89255899	Glycyl-tRNA synthetase alpha chain	
LSQNNFQIESL	11	Kb	101	0.2	89255900	UDP-N-acetylmuramoylalanyl-D-glutamate--2,6- diaminopimelate ligase	
LNSYNPNNL	9	Kb	397	0.2			
AMQELLQQTSL	11	Kb	445	0.1	89255904	aspartate semialdehyde dehydrogenase	
KVVINKSMDEL	11	Db	95	0.2	89255905	threonine synthase	
AQYFAGERL	9	Kb	109	0.3	89255909	conserved hypothetical protein	
HAAVNMLPV	9	Db	87	0.3	89255912	Carbon-nitrogen hydrolase family protein	
VMNVNPISFIL	11	Db	226	0.3	89255923	ABC transporter, membrane protein	
KSYNPDFAYL	10	Kb	111	0.2	89255935	Type III restriction enzyme	
TMLCFSSL	8	Kb	41	0.3	89255940	DNA gyrase, subunit A	
AGIKTIHYVSP	11	Kb	493	0.2	89255947	Lipid-A-disaccharide synthase	
HIYSSIFITL	10	Kb	218	0.7	89255953	3-Deoxy-D-manno-octulosonic-acid transferase	
NTYFSKLKSL	10	Kb	127	0.7	89255965	Cyclopropane-fatty-acid-phospholipid synthase	
ISYGPTMFGQL	11	Kb	57	0.7	89255979	sugar transport protein	
VYISETSV	8	Kd	69	0.4			
NVFISYGPTM	10	Kb	295	0.4			
TTLMLSRLL	9	Kb	348	0.3			
SAPKHVEQL	9	Kb	91	NO	89255986	isocitrate dehydrogenase	
DNYLSQPMIYL	11	Kb	192	0.3	89255988	ATp-dependent helicase	
FYGKVTFSL	9	Kd	22	0.5	89255990	dTDP-glucose 4,6-dehydratase	
FFFFFLSMIYL	11	Kb	253	0.4	89255996	Membrane protein/O-antigen protein	
VGLFTKLTIIL	11	Kb	98	1.1	89255996	asparagine synthase	
FGIQNGIL	8	Db	161	0.2			
RYFYPSLDSL	10	Kd	297	0.7	89255999	sugar transamine/perosamine synthetase	
TVFQTWLRL	9	Kb	118	0.4	89256001	O-antigen flippase	**M**
IYNTYAGL	8	Kb	59	0.5	89256002	Glycosyltransferase	
NDPHRYGIVEF	11	Dd	106	0.2	89256003	Glucose-1-phosphate thymidylyltransferase	
YSGYTNTEIF	10	Dd	319	0.4	89256005	Transposase	
SAMTDKICWL	10	Db	262	0.2	89256007	Phosphomannomutase	
SYFASPDDIYV	11	Kd	184	0.4	89256007	transcription termination factor Rho	
VTLTNQDI	8	Db	425	0.3			
AIFWLSVML	9	Kb	106	0.5	89256012	ATPase, AAA family	
IRFSKAIEELL	11	Kb	363	0.2	89256018	ABC transporter, membrane protein	
FTFVLYYL	8	Kb	70	0.6	89256018	NADH oxidase	
AGPANKQGRL	10	Kb	379	0.2			
YIMISHYYDTI	11	Db	158	0.5	89256027	conservered hypothetical membrane protein	
SILAVPLL	8	Kb	325	0.2			
KQYGYPIV	8	Kb	120	0.2	89256038	hypothetical protein	
IYTSIAEASLL	11	Kd	323	0.5	89256054	conservered hypothetical membrane protein	
ISLAMSSLVLL	11	Kb	77	0.6	89256054	conserved hypothetical membrane	
MVILLSLSL	9	Kb	416	0.8			
SVPFRVIAML	10	Kb	80	0.5	89256056	Exodeoxyribonuclease V gamma chain	
QFKPINSVL	9	Kd	259	0.1	89256066	DNA ligase	
LAYQTQRRSIV	11	Kb	278	0.3	89256068	Transposase	
SFFKITLPL	9	Kb	409	0.4	89256070	polyamine transporter, subunit H, ABC transporter, membrane protein	
SAAMAYLPTM	10	Kb	89	0.8	89256077	major facilitator superfamily (MFS) transport protein	
YYLAINMGGL	10	Kd	30	0.7	89256080	Proton-dependent oligopeptide transport (POT) family protein	
YQFLNPLWIL	10	Db	91	0.8			
KMLTVATFEYI	11	Db	141	0.3	89256083	hypothetical protein	
TYGIVTNL	8	Kd	131	0.3	89256090	FAD binding family protein	
LYADANTL	8	Kd	275	0.3	89256091	hypothetical protein	
AGFSAFSGI	9	Kb	472	0.1	89256092	serine hydroxymethyltransferase	
ISQILSKTIAL	11	Kb	223	0.5	89256094	hypothetical membrane protein	
LPFKYVTL	8	Kb	141	0.7	89256096	glycosyl transferase family 8 protein	
SYASKYSII	9	Kd	38	0.2	89256099	choloylglycine hydrolase family protein	
TVIKDLNNNKL	11	Kb	442	0.6			
KYIKENGV	8	Kd	60	0.3	89256104	thymidylate synthase	
SYPGILSVTFL	11	Kd	222	0.3	89256107	cysteine desulfarase	

Sequence	Length	Predicted allele	Affinity (IC$_{50}$, nM)	Cluster density	Protein gi#	Protein annotation	Responder
KYLSIETSL	9	Kd	1	0.3	89256116	monooxygenase family protein	
FALALMYAI	9	Db	57	0.5	89256124	hypothetical membrane protein	
ILLLDDMFISL	11	Kb	219	0.2	89256127	hypothetical membrane protein	
KYRLEISYI	9	Kd	230	0.2			
AIYQSDFMSM	10	Kb	53	0.3	89256131	Exodeoxyribonuclease I	
QDYLNKIVSL	10	Kb	297	0.5			
LFNCLITPL	9	Kd	103	0.3	89256144	SsrA (tmRNA)-binding protein	
LGPVFRGL	8	Kb	28	NO	89256145	conserved hypothetical protein	
VNYSVYGL	8	Kb	10	0.4	89256148	VacJ lipoprotein	
AALRNAYI	8	Db	35	0.4			
EYLRNHNKCPI	11	Kd	27	0.1	89256150	conserved hypothetical protein	
IKIQMQQRQKL	11	Kb	194	0.2	89256157	hypothetical lipoprotein	
AAFNLPIIEL	10	Kb	77	NO	89256159	N-acetylglucosamine-6-phosphate deacetylase	
LSLQKQSKNEL	11	Kb	70	0.3	89256172	3-dehydroquinate synthetase	
YVLDYLANL	9	Kb	369	0.4	89256175	bifunctional proline dehydrogenase,pyrroline-5-carboxylate dehydrogenase	
DIPNNSQF	8	Dd	315	0.2	89256176	amino acid transporter family protein	
LGLGYISFLL	10	Kb	345	0.4			
SNFIFLYGI	9	Kb	355	0.9	89256177	major facilitator superfamily (MFS) transport protein	
SYQQSMAHINL	11	Kd	35	0.3	89256178	bifunctional 4-phosphopantothenoylcysteine decarboxylase	
INPYYDNKF	9	Dd	362	0.2	89256184	Ferrochelatase	
FYCGGGFRSAL	11	Kd	7	0.3	89256194	Rhodanese-like family protein	
IIYCWLIL	8	Kb	194	0.2	89256195	aromatic amino acid transporter of the HAAAP family	
RSIPYIIL	8	Kb	46	0.4	89256197	D-methionine binding transport protein, ABC transporter	
LILLYPLTRL	10	Kb	186	0.7			
VAIENSYTF	9	Db	85	0.4	89256201	hypothetical lipoprotein	
TIHNLTFYQNL	11	Kb	283	0.3	89256203	queuine tRNA-ribosyltransferase.	
TVMRNRINAL	10	Kb	67	0.3	89256208	preprotein translocase, subunit D, membrane protein	
KYTTVFSVL	9	Kd	3	0.4	89256209	preprotein translocase, subunit F, membrane protein	
TFMTLAAI	8	Kd	242	0.4	89256212	3-phosphoshikimate 1-carboxyvinyltransferase	
ANYHTDSSL	9	Kb	132	0.2	89256218	conserved hypothetical protein	
FHYWIKPL	8	Kb	324	0.1			
KYICIVCGLI	10	Kd	14	0.3	89256219	Rubredoxin	
IGVDESKYNLL	11	Kb	208	NO	89256220	hypothetical protein	
TFAPTMGTTL	10	Kd	35	0.5	89256223	Sodium-dicarboxylate symporter family protein	
FMIIFFSAM	9	Kb	43	0.4	89256225	major facilitator superfamily (MFS) transport protein	
YSVESYNFLL	10	Db	244	0.5			
SYNFLLFSRLI	11	Kd	442	0.6			
LSYANDTYFDL	11	Kb	54	0.5	89256229	hypothetical protein	
SYKSTLELL	9	Kd	44	0.6	89256235	Peptidase, M24 family protein	
ESYHFPICSV	10	Kb	494	0.3	89256237	beta-lactamase	
VSGQFSPKSIM	11	Kb	372	0.3	89256239	hypothetical protein	
SQIMIHQPL	9	Kb	101	0.4	89256249	ATP-dependent Clp protease subunit P	
LLLSYLYEEL	10	Kb	242	0.5	89256251	DNA-binding, ATP-dependent protease La	
LPPSTNIHL	9	Dd	181	0.5	89256268	Transcription-repair coupling factor,ATP-dependent	
INKIFYLSFL	10	Kb	422	0.3	89256270	hypothetical membrane protein	
IIYDYCEF	8	Kb	171	NO	89256270	Endonuclease III	
IIYFGWRFKFI	11	Kb	365	0.8			
ISINLGSLL	9	Kb	308	0.8	89256276	Proton-dependent oligopeptide transport (POT) family protein	
KYTTDHGFYAV	11	Kd	243	NO	89256278	lipoic acid synthetase	
ASYSVAFLWL	10	Kb	141	0.4	89256282	major facilitator superfamily (MFS) transport protein	
TQLREQNNSL	10	Kb	105	0.2	89256284	DNA recombination protein RmuC family protein	
TYKSKYSPF	9	Dd	236	0.1	89256288	hypothetical protein	
AFLPLKNDYI	10	Kd	174	0.2	89256289	histidine decarboxylase	
LNFYYLIANV	10	Kb	339	0.6	89256293	nicotinamide mononucleotide transport (NMT) family protein	
FSFVLISM	8	Kb	169	0.4	89256294	Sodium/hydrogen exchanger family protein	
EYIDLSNSI	9	Kd	130	0.4	89256300	conserved hypothetical membrane protein	
AKADFYTRL	9	Kb	207	0.3	89256304	GTP-binding protein	
TLFKKASRL	9	Kb	331	0.6	89256312	Proton-dependent oligopeptide transport (POT) family protein	
CSILKQYIAL	10	Kb	35	0.7	89256313	ATP-dependent protease, ATP-binding subunit	
VYPNRRNDIVL	11	Dd	120	0.4	89256316	Lipoate-protein ligase A	
KYQIKNTM	8	Kd	92	0.2	89256320	FAD linked oxidase	
VAYSFGDMNL	10	Kb	34	0.2	89256320	Ribonucleoside-diphosphate reductase, alpha subunit	
KYNRLWDV	8	Kd	45	0.3			
GKHYTFESL	9	Kb	337	0.4	89256338	hypothetical protein	
IHINAWFNVL	10	Kb	460	0.1	89256339	haloacid dehalogenase	
AAYPTVRAI	9	Kb	469	0.3	89256356	cytidylate kinase	
VVPTSIIQQL	10	Kb	343	0.3	89256357	phosphoserine aminotransferase	
FSKEYYKL	8	Kb	51	0.3	89256360	hypothetical protein	
IYFIIFLPQTI	11	Kd	327	0.7	89256377	hypothetical membrane protein	
SNFRFTTSI	9	Kb	109	0.2	89256384	D-alanyl-D-alanine carboxypeptidase (Penicillin binding protein)	
EGYMDVVGL	9	Kb	238	0.2	89256387	DNA primase	
STILSYPIVI	10	Db	111	0.5	89256388	RNA polymerase sigma-70 factor	
GLVTNVLTL	9	Db	312	0.5	89256391	putative alpha-xylosidase	
INPYLVKDYPL	11	Kb	118	0.4	89256392	putative alpha-xylosidase	
SGYILTKAKTM	11	Kb	33	0.2	89256402	conserved hypothetical protein	
SGLIGHSELM	10	Kb	375	0.2	89256409	GDSL-like Lipase/Acylhydrolase family protein	
SSIYFIKGL	9	Kb	21	0.4	89256411	short chain dehydrogenase family protein	
TARGRIATL	9	Kb	415	0.3	89256412	holliday junction DNA helicase, subunit B	
GYITNLAEL	9	Kd	12	0.5	89256424	rRNA methyltransferase	
TILRTAIAVGL	11	Kb	315	0.2			
CAFLIADGVL	10	Kb	208	NO	89256431	Alanyl-tRNA synthetase	
LNQYYQFL	8	Kb	391	0.9	89256432	conserved hypothetical protein	
LIFLNLVYFTL	11	Kb	48	0.9	89256438	conserved hypothetical membrane protein	
IYVYLLYSWEL	11	Kd	498	0.7			
CTFENKKNSL	10	Kb	29	NO	89256439	isochorismatase hydrolase family protein	
FIYQEYQKYL	10	Kb	58	0.3	89256441	ATP-dependent DNA helicase	
ISFWKSAL	8	Kb	172	0.3			
AQPISISIPL	10	Kb	214	0.2	89256450	transcription regulator	
KYEEGMTEFEL	11	Kd	47	0.2	89256452	Transposase	
HAVSQYWL	8	Db	245	0.3	89256453	hypothetical protein	
VTVKMIRVM	9	Kb	403	0.4	89256458	conserved hypothetical membrane protein	
GALIPIFAAYL	11	Kb	392	0.3	89256459	conserved hypothetical membrane protein	
CAMSAILGL	9	Kb	303	0.1	89256464	malonyl coA-acyl carrier protein transacylase	
IKYSIKAKLQL	11	Kb	163	0.4	89256468	conserved hypothetical protein	

Sequence	Length	Predicted allele	Affinity (IC_{50}, nM)	Cluster density	Protein gi#	Protein annotation	Responder
QYIKQMWSF	9	Kd	331	0.3	89256475	Trehalase, pseudogene	
IAFDEQDLERL	11	Kb	85	NO	89256477	conserved hypothetical protein	
IYQQTFSNTI	10	Kd	11	0.2	89256483	conserved hypothetical protein	
KEYFPEIFFPL	11	Kb	387	0.1	89256486	conserved hypothetical protein	
SMLLQDVEM	9	Db	65	0.3	89256488	conserved hypothetical protein	
FIPTNPTTLL	10	Db	220	0.5	89256489	conserved hypothetical protein	
GIYLSTRIKTL	11	Kb	282	0.4			
KSYYMNPTLI	10	Kb	226	0.9	89256490	conserved hypothetical protein	
GALKLYLLAM	10	Kb	320	0.2	89256493	conserved hypothetical protein	
YSSTYKNI	8	Kb	428	0.3			
FYINGRVTI	9	Kd	3	0.3	89256494	conserved hypothetical protein	
KALSAIEL	8	Db	392	0.2	89256495	conserved hypothetical membrane protein	
IYLRMYVEASI	11	Kd	7	0.2	89256498	tRNA modification GTPase trmE family protein	
VGYTNQNESI	10	Kb	115	0.3			
AVYTTAKYAGM	11	Kb	81	0.3	89256504	Sodium-solute symporter family protein	
STIVGIVSAL	10	Kb	469	0.4			
TPYQINSWSL	10	Kb	212	0.2	89256520	Regulatory protein	
KSLADIAYI	9	Db	366	0.4	89256521	Cobalamin (vitamin B12) synthesis protein/P47K family protein	
SSLILINL	8	Kb	89	0.2	89256526	hypothetical protein	
LLYARFFHKLM	11	Kb	117	0.3	89256533	Leucyl-tRNA synthetase	
KYIGGIEHAIM	11	Kd	237	0.2			
RNYYKLVQL	9	Kb	51	0.3	89256534	hypothetical protein	
KYEIDKSLI	9	Kd	184	0.6			
YYNHLIVCL	9	Kd	467	0.4	89256539	hypothetical protein	
SSFIIAYV	8	Kb	250	0.5	89256541	Amino-acid permease	
LHQPIRTKI	9	Kd	227	0.1	89256543	conserved hypothetical protein	
ESWKYTNI	8	Kb	90	0.2	89256547	conserved hypothetical protein	
LQLMMLEPKL	10	Kb	116	0.2	89256548	ABC transporter, ATP-binding protein	
SLILFLTL	8	Kb	381	0.5	89256553	hypothetical protein	
KYLFFELIIFI	11	Kd	191	0.3	89256555	conserved hypothetical membrane protein	
HYAIGASLVAI	11	Kd	38	0.4	89256555	hypothetical protein	
TYVLVCIIL	9	Kd	143	0.4			
FLLSNEASFII	11	Db	272	0.2	89256563	Short-chain dehydrogenase	
ALYCAISGHRM	11	Kb	411	0.3	89256566	Pyruvate/2-oxoglutarate dehydrogenase complex	
LVFSDAEAM	9	Kb	224	0.3	89256569	Proton-dependent oligopeptide transport (POT) family protein	
QYHPEAYL	8	Kd	188	0.5	89256573	anthranilate synthase component II	
VSDDFLEVHL	10	Kb	362	NO	89256579	conserved hypothetical protein	
STYQEIDLL	9	Kb	42	0.2	89256580	Adenosylmethionine-8-amino-7-oxononanoate aminotransferase	
VSFVTNIL	8	Kb	72	0.3	89256587	major facilitator superfamily (MFS) transport protein	
GVYLPKYSAEL	11	Kb	453	0.1	89256587	hypothetical membrane protein	
IIYFLAIF	8	Kb	487	0.4			
QYQSDTQGNI	10	Kd	77	0.3	89256595	fatty acid hydroxylase	
SKLLNFNLKSI	11	Kb	424	0.3			
FYYPAVLIFL	10	Kd	165	0.7	89256605	amino acid antiporter	
YYGKYRSI	8	Kd	226	0.4	89256610	Transposase	
VSQQFRSYEL	10	Kb	142	0.3	89256613	Glutamate--cysteine ligase	
SVLVLVKSM	9	Kb	277	0.3			
DTALFVEAM	9	Kb	497	0.3			
LYAQNKTTI	9	Kd	3	0.6	89256614	virulence factor MviN	
YVLFNGVMGVI	11	Db	221	0.4			
SGDKYAFV	8	Kb	281	0.6			
TYLAMLGYLM	10	Kd	472	0.5			
LGFDITYYAI	10	Kb	401	0.3	89256623	hypothetical membrane protein	
YYFINSRIYSL	11	Kb	356	0.2	89256624	hypothetical protein	
TFYDCLNIL	9	Kb	256	0.3	89256625	Transposase	
FELENWCQI	9	Db	289	0.4	89256626	hypothetical protein	
VQYNQLVGRV	10	Kb	478	0.5	89256632	outer membrane associated protein	
VVLHEIAHL	9	Kb	337	0.3	89256639	conserved hypothetical protein	
QALSQCANSL	10	Kb	204	0.2	89256640	prephenate dehydratase	
IGLPIAVFGM	10	Kb	432	0.5	89256645	conserved hypothetical membrane protein	
KYKQRKEHERL	11	Kd	234	0.1	89256650	Transposase	
FFVRQPEAL	9	Kd	498	0.6	89256656	UTP--glucose-1-phosphate uridylyltransferase	
HYVPGGFKDFM	11	Kd	83	0.3	89256658	hypothetical membrane protein	
AVTMFFSANM	10	Kb	345	0.4	89256666	conserved hypothetical membrane protein	
MSLDLDKPM	9	Kb	353	0.3			
TTIKLTRPSL	10	Kb	49	0.2	89256671	hypothetical lipoprotein	
ISLTNIII	8	Db	36	0.5	89256687	Galactose-proton symporter, MFS transport protein	
FSVIAFFAFL	10	Db	103	0.6			
CYLCPTNI	8	Kd	41	NO	89256689	Galactose-1-phosphate uridylyltransferase	
YSIYNRGEIM	10	Db	96	0.5	89256693	conserved hypothetical protein	
TSFYKKYAKSL	11	Kb	16	0.3	89256709	capsule biosynthesis protein capB	
MALQPLLQSL	10	Kb	70	0.3			
WGLLSDYL	8	Db	200	0.4	89256710	major facilitator superfamily (MFS) transport protein	
VAINPGSSQL	10	Kb	462	0.1	89256713	Carbohydrate/purine kinase pfkB family protein	
SYAKEDSRI	9	Kd	81	0.2	89256717	glycosyl transferase family protein	
MSLIRKIKLSL	11	Kb	101	0.4	89256718	hypothetical protein	
PYPYADLLQTI	11	Dd	218	0.5	89256722	glycosyl transferases group 1 family protein	
LVLSHFLGKGL	11	Kb	354	0.4	89256732	Arylsulfatase	
SNPKYLGV	8	Kb	39	0.2	89256745	50S ribosomal protein L27	
LGPMFGPL	8	Kb	55	0.4	89256759	major facilitator superfamily (MFS) transport protein	
GFCFGMFTMI	10	Kd	220	0.5	89256761	hypothetical protein	
SYSSGDILL	9	Kd	242	NO	89256766	thiamine pyrophospokinase	
FTLSEDDLFL	10	Db	492	0.3			
LCTNMFINTL	10	Kb	288	0.5	89256769	Permease YjgP/YjgQ family protein	
KYAKLLPSVVV	11	Kd	427	0.3			
INLYISASL	9	Kb	47	0.7	89256774	conserved hypothetical membrane protein	
SAVLREWLNQL	11	Kb	154	0.3	89256778	FAD-binding family protein	
ASYFIFCILMV	11	Kb	301	0.7	89256782	polysaccharide biosynthesis protein (export protein)	
KTVTNSQDKV	10	Db	415	NO	89256783	hypothetical protein	
KYKSGDLL	8	Kd	82	0.2	89256785	ABC transporter, ATP-binding and membrane protein	
LTTQLYMFL	9	Kb	317	0.9	89256790	major facilitator superfamily (MFS) transport protein	
RYFEGRDTPI	10	Kd	8	0.4	89256796	D-alanyl-D-alanine carboxypeptidase/D-alanyl-D-alanine-endopeptidase	
VILALYSAM	9	Kb	43	0.4			
STYYLVNV	8	Kb	127	0.5	89256797	Glycerol-3-phosphate transporter	

Sequence	Length	Predicted allele	Affinity (IC_{50}, nM)	Cluster density	Protein gi#	Protein annotation	Responder
LKIFARLHSL	10	Kb	265	0.2	89256800	ABC transporter, ATP-binding protein	
YAIFLKEVIHM	11	Db	169	0.5	89256801	ABC-2 type transport system permease protein	
RYYAIFLKEVI	11	Kd	226	0.5			
ATFPFIIANL	10	Kb	52	0.4	89256802	ABC transporter, membrane protein	
KSVQNPDLQEV	11	Db	93	0.2	89256803	Transposase	
VLYFFAIFLNM	11	Kb	56	0.3	89256809	serine transporter	
VGLIFIVL	8	Kb	173	0.7			
ASLMAISTL	9	Kb	59	0.3	89256813	major facilitator superfamily (MFS) transport protein	
AYGLGSVISPV	11	Kd	399	0.4			
IYLLNALYGYI	11	Db	65	0.7	89256814	nicotinamide mononucleotide transport (NMT) family protein	
SSFIETLSI	9	Kb	478	0.4	89256815	23S rRNA (Uracil-5-)-methyltransferase	
IMLFNDRKL	9	Db	145	0.3	89256818	uridine kinase	
IRVRLFESL	9	Kb	107	0.4	89256824	hypothetical protein	
WYITGDKGKI	10	Kd	18	0.3	89256826	conserved hypothetical protein	
ILPIFHAFGL	10	Kb	270	0.2			
YSPKLLEMSL	10	Kb	166	0.7	89256836	hypothetical membrane protein	
LTFHRAIDL	9	Kb	242	NO	89256843	CutC family protein	
IGPELMEKL	9	Kb	159	NO	89256848	thioredoxin reductase	
SIYYLVAGIFI	11	Kb	482	0.5	89256852	hypothetical membrane protein	
KYIYTSDDNM	10	Kd	67	0.4	89256859	Putrescine-binding periplasmic protein	
LMFITAVKL	9	Kb	113	0.6	89256860	Glutamate:gamma-aminobutyric acid antiporter family protein	
CALLTAEM	8	Db	173	0.6			
SGVNMFRNHL	10	Kb	68	0.4	89256861	PerM family protein	
IAILKKNGI	9	Kb	360	NO	89256862	Single-stranded-DNA-specific exonuclease	
SAYIGMIKL	9	Kb	47	0.5	89256866	Transposase	
VSILNKIDIL	10	Db	53	0.3	89256867	hypothetical membrane protein	
IEAINYINM	9	Kb	474	0.6	89256870	3-dehydroquinate dehydratase, type II	
GDPYDNAWK	9	Dd	67	NO	89256874	organic solvent tolerance protein	
ITYPEFTEL	9	Kb	64	0.4	89256886	Dolichyl-phosphate-mannose-protein mannosyltransferase	
KYEEGMTEF	9	Kd	298	0.2	89256887	Transposase	
TRYASILRQRL	11	Kb	170	0.4	89256893	phosphoenolpyruvate carboxykinase	
TKYSYAGT	8	Kb	189	0.5	89256900	conserved hypothetical membrane protein	
KSIKKLFI	8	Db	198	0.5	89256901	major facilitator superfamily (MFS) transport protein	
TGIALYETV	9	Kb	374	0.2	89256903	rRNA methylase	
PLYSFAYL	8	Kb	95	0.6	89256905	hypothetical protein	
VAFQELDAI	9	Kb	181	0.4	89256906	hypothetical membrane protein	
ASAKQIANL	9	Kb	319	0.2			
LSILRGWSL	9	Kb	299	0.1	89256907	Transposase	
VKSIFIASCSM	11	Kb	470	0.6	89256910	hypothetical protein	
SNWQRVRIDNL	11	Kb	310	NO	89256912	Chitinase, fragment	
YLIINFYGVKL	11	Db	493	0.8	89256917	amino acid transporter protein, fragment	
ASFHKSSKL	9	Kb	250	NO	89256920	Transposase	
YSVHYAGL	8	Kb	14	0.4	89256922	major facilitator superfamily (MFS) transport protein	
WAGLLYTPTVI	11	Db	355	0.3	89256924	major facilitator superfamily (MFS) transport protein	
VWYYFNKICQP	11	Kb	371	0.4			
LAQAYFIFNM	10	Kb	339	0.4	89256927	hypothetical protein	
IGINIINYV	9	Kb	377	0.3	89256935	nucleoside permease NUP family protein	
TYEFLNDQV	9	Kd	476	0.3	89256935		
PVIQIIASL	9	Kb	218	0.4	89256941	Lipid A transport protein, ABC transporter	
SNCIFIFNTL	10	Kb	280	0.3	89256943	disulfide bond formation protein	
IVVYAPIGMM	10	Kb	447	0.5	89256945	Transporter AcrB/AcrD/AcrF family	
ANYWLHSGM	9	Kb	42	0.2	89256953	Cysteinyl-tRNA synthetase	
VFLLTYDEL	9	Kb	334	0.5	89256954	Adenine-specific methylase, HemK family	
IAYRQQPVKL	10	Kb	16	0.3	89256962	hypothetical protein	
DYVGKFSI	8	Kd	368	0.2	89256964	hypothetical protein	
VSDLNVSYAL	10	Kb	434	0.3			
ITLDNLLTI	9	Db	70	0.8	89256966	metal ion transporter	
LEYQKYKQSL	10	Kb	22	0.2	89256969	Sodium/proline permease	
RAYIKQGKSL	10	Kb	111	0.5	89256972	amino acid transporter	
SFLIPYNNPSL	11	Kb	174	0.3			
KAIKQTFWRL	10	Db	410	0.3			
TAFSTIATTL	10	Kb	38	0.2	89256977	potassium uptake protein	
AYLSGRNELL	10	Kd	37	0.5	89256978	hypothetical protein	
IIYQNENYAP	10	Kb	312	0.4	89256979	ProP osmoprotectant transporter, fragment	
IIISLIAML	9	Kb	119	0.7	89256985	conserved hypothetical membrane protein	
DHPDASTL	8	Dd	424	0.2	89256987	Transposase	
LGYKYFWNQKL	11	Kb	45	0.3	89256989	modification methylase	
VNFGMFCTL	9	Kb	51	0.3	89256994	Eflux protein	
SILTKILFVSL	11	Kb	139	0.6	89256999	hypothetical protein	
IGYLLISKV	9	Kb	165	0.7			
VALIAIPWYAM	11	Kb	99	0.4	89257009	fatty acid desaturase	
IGLFLDMPL	9	Kb	54	0.4	89257012	DNA-directed RNA polymerase, beta subunit	
FGPEKLDDF	9	Dd	272	0.2	89257015	50S ribosomal protein L10	
TAIQGYTI	8	Db	151	0.2	89257021	Helicase	
LYVAMTRARKL	11	Kd	478	0.5			
IGVKLYEFL	9	Kb	67	0.2	89257025	anaerobic glycerol-3-phosphate dehydrogenase	
GIFASKVFESL	11	Kb	422	0.3	89257029	hypothetical protein	
SYQEENNI	8	Kd	122	0.4	89257031	sensor histidine kinase	
MGILFPLL	8	Kb	12	0.6	89257033	cytochrome oxidase bd-II, subunit II	
AVFRRLENTI	10	Kb	332	0.2	89257036	twitching motility protein PilT	
LAGYNVSAL	9	Kb	162	0.4	89257045	adenine phosphoribosyltransferase	
PGIMFRDITPL	11	Kb	237	0.2			
IAIPLCVVAM	10	Kb	213	0.3	89257051	succinate dehydrogenase, cytochrome b556	
FAMSIAVFFL	10	Db	85	0.2	89257064	ATP synthase A chain	
FAASFLATAAM	11	Kb	269	0.4	89257066	major facilitator superfamily (MFS) transport protein	
FSLLEIKL	8	Db	182	0.2	89257067	ribosomal large subunit pseudouridine synthase C	
QYLKPYHAEL	10	Kd	86	0.2	89257070	Histidyl-tRNA synthetase	
VIYEGTLESL	10	Kb	41	0.2	89257072	translation initiation factor IF-2	
TFYDCLNIL	9	Kb	256	0.3	89257076	Transposase	
FSPVTLMCSLL	11	Db	327	0.5	89257079	NADH dehydrogenase I, L subunit	
YAFVMMVGVLL	11	Kb	488	0.6			
AAMTAAITLTL	11	Db	352	0.4	89257081	NADH dehydrogenase I, J subunit	
ISYGEITFSI	10	Kb	196	0.2	89257088	NADH dehydrogenase I	
ATMMIYSSL	9	Kb	31	0.4	89257095	conserved membrane protein	

Sequence	Length	Predicted allele	Affinity (IC_{50}, nM)	Cluster density	Protein gi#	Protein annotation	Responder
FALSLEFL	8	Db	98	0.4			
FATMMIYSSL	10	Kb	188	0.4			
KALGMIVYGPL	11	Kb	284	0.4			
KNYFYPDL	8	Kb	9	0.5	89257101	Aspartyl/glutamyl-tRNA(Asn/Gln) amidotransferase subunit B	
MQLKSFEML	9	Kb	170	0.3	89257105	RND efflux membrane fusion protein	
CIVLVIFVPM	10	Kb	228	0.4	89257107	toxin secretion ABC transporter ATP-binding protein	
KYAKMLEVEL	10	Kd	10	0.2	89257110	adenylosuccinate lyase	
TQYLYKRT	8	Kb	160	0.5	89257113	hypothetical lipoprotein	
QMLAQLKSL	9	Kb	273	0.4	89257115	phosphoribosylformylglycinamidine synthase	
TYIKDITPVI	10	Kd	126	0.2	89257122	protease yegQ	
MLPSGSREII	10	Dd	346	NO			
IAMLFPATSSL	11	Kb	22	0.4	89257123	multidrug resistance protein, membrane located	
VVFGSAGL	8	Kb	313	0.4			
SYSIVKTHF	9	Kd	105	0.2	89257125	amino acid transporter	
SAFIFAWPTL	10	Kb	20	1.2	89257127	aromatic amino acid transporter of the HAAAP family	
SIIYLLLLYSL	11	Kb	78	0.5			
FAIAVVIM	8	Db	390	0.5			
IGISGMDRL	9	Kb	416	0.2	89257133	Potassium-transporting ATPase B chain	
TAMTFSYV	8	Kb	213	0.4	89257144	hypothetical protein	
SSIIPTYFLLL	11	Kb	298	0.9	89257149	conserved hypothetical membrane protein	
QSFNALLKTL	10	Kb	232	0.3	89257153	DNA polymerase III, gamma/tau subunits	
FAVINMYVYL	10	Db	5	0.5	89257168	YggT family protein	
IAPSYTQEAL	10	Kb	25	0.3	89257172	bifunctional purine biosynthesis protein	
FFITTIICIL	10	Kd	190	0.5	89257175	permease of ABC transporter	
ESLQNFLFENM	11	Kb	192	0.4	89257180	tRNA processing ribonuclease BN	
ISHKMTSL	8	Kb	240	0.6			
MQYMKVTL	8	Kb	403	0.6			
NVYRYSLL	8	Kb	9	0.3	89257182	Primosomal protein N	
RILDKPISL	9	Kb	443	0.3			
LYIPAFIAII	10	Kd	54	0.8	89257187	major facilitator superfamily (MFS) transport protein	
IAQYRCINAL	10	Kb	71	0.5			
NPYQALAQL	9	Kb	446	0.3	89257198	conserved hypothetical protein	
MGASFRNV	8	Kb	394	NO	161353781	Transaldolase	
